# Establishing a prognostic model of ferroptosis- and immune-related signatures in kidney cancer: A study based on TCGA and ICGC databases

**DOI:** 10.3389/fonc.2022.931383

**Published:** 2022-08-26

**Authors:** Zhijun Han, Hao Wang, Jing Long, Yanning Qiu, Xiao-Liang Xing

**Affiliations:** ^1^ Department of Urology, Department of Ultrasonography, Zhuzhou Hospital Affiliated to Xiangya School of Medicine, Central South University, Zhuzhou, China; ^2^ Hunan Provincial Key Laboratory for Synthetic Biology of Traditional Chinese Medicine, Hunan University of Medicine, Huaihua, China; ^3^ Department of Urology, The First Affiliated Hospital to Hengyang Medical School, South China University, Hengyang, China; ^4^ First College for Clinical Medicine, Xinjiang Medical University, Urumqi, China

**Keywords:** kidney cancer, immune, ferroptosis, overall survival, prognosis

## Abstract

**Background:**

Kidney cancer (KC) is one of the most challenging cancers due to its delayed diagnosis and high metastasis rate. The 5-year survival rate of KC patients is less than 11.2%. Therefore, identifying suitable biomarkers to accurately predict KC outcomes is important and urgent.

**Methods:**

Corresponding data for KC patients were obtained from the International Cancer Genome Consortium (ICGC) and The Cancer Genome Atlas (TCGA) databases. Systems biology/bioinformatics/computational approaches were used to identify suitable biomarkers for predicting the outcome and immune landscapes of KC patients.

**Results:**

We found two ferroptosis- and immune-related differentially expressed genes (FI-DEGs) (*Klotho* (*KL*) and *Sortilin 1* (*SORT1*)) independently correlated with the overall survival of KC patients. The area under the curve (AUC) values of the prognosis model using these two FI-DEGs exceeded 0.60 in the training, validation, and entire groups. The AUC value of the 1-year receiver operating characteristic (ROC) curve reached 0.70 in all the groups.

**Conclusions:**

Our present study indicated that *KL* and *SORT1* could be prognostic biomarkers for KC patients. Whether this model can be used in clinical settings requires further validation.

## Introduction

Kidney cancer (KC) is the second most common cancer of the urinary system. Statistical data from the Global Cancer Statistics 2020 report showed that there were over 430,000 new cases and 180,000 deaths related to KC (1). KC remains one of the most challenging cancers in urology despite the availability of various therapeutic approaches, such as surgery, chemotherapy, radiotherapy, and recently proposed immunotherapy (2). The main reasons for this phenomenon are delayed diagnoses and high metastasis rates. For example, almost one-third of people diagnosed with KC have advanced KC (3, 4). Approximately 40% of patients with advanced cancer eventually develop metastasis (3, 4), and the 5-year survival rate of these patients is less than 11.2% (3, 4). Therefore, it is important to identify suitable biomarkers to predict KC outcomes.

More evidence shows that carcinogenesis is not only related to cancer cells but also to the microenvironment (5–7). Previous studies have demonstrated that anticancer effects can be achieved by inducing immunosuppressive cells, such as regulatory T cells and myeloid-derived suppressor cells, to infiltrate the tumor microenvironment and mediate immune dysfunction (8). Recent studies have indicated that ferroptosis, a form of iron-dependent programmed cell death, is involved in the progression of several cancers (9–11). The kidney is involved in iron metabolism. Several studies have shown that clear cell renal cell carcinomas (RCCs) are closely related to iron metabolism (12, 13). RCC is an immunogenic tumor. Ferroptosis regulation can inhibit the migration and invasion of clear cell RCC cells (14, 15). Additionally, previous studies have indicated that immunity and ferroptosis can regulate each other to achieve their anticancer effects (16–18). Therefore, we identified suitable biomarkers for predicting KC patient prognosis and immune status by conducting an integrated study of ferroptosis- and immune-related genes.

## Materials and methods

### Data collection and preprocessing

We obtained RNA sequence information for 91 KC and 45 normal tissues and their clinical information from the ICGC database, and 818 KC and 104 normal tissues and their corresponding clinical information from TCGA database (**Table 1**). In our present study, ICGC data, TCGA data, and the ICGC and TCGA merge data were set as training, validation, and entire groups, respectively. Ferroptosis- and immune-related genes were obtained from the FerrDb and ImmPort, respectively. We performed differential expression analyses using the package “DESeq2” in R (3.6.2) to identify differential expression genes (DEG) between KC patients and normal tissues. The threshold values were set to ≥200 for baseMean, ≥1 for |log2 fold change (FC)|, and<0.05 for adj *p* value.

**Table 1:** Clinical features of patients with kidney cancer.

Variables	Training group (*n* = 91)		Validation group (*n* = 818)	
	No.	%	No.	%
Vital				
Alive	61	67.03%	217	26.53%
Death	30	32.97%	601	73.47%
Gender				
Female	39	42.86%	262	32.03%
Male	52	57.14%	556	67.97%
Age				
≤60	46	50.55%	400	48.90%
>60	45	49.45%	408	49.88%
Tumor (T)				
T1	54	59.34%	464	56.72%
T2	13	14.29%	101	12.35%
T3	22	24.18%	238	29.10%
T4	2	2.20%	13	1.59%
X	0	0.00%	2	0.24%
Nodes (N)				
N0	79	86.81%	288	35.21%
N1	2	2.20%	40	4.89%
N2	0	0.00%	4	0.49%
NX	10	10.99%	486	59.41%
Metastasis (M)				
M0	81	89.01%	515	62.96%
M1	9	9.89%	87	10.64%
MX	1	1.10%	216	26.41%
Stage				
I	53	58.24%	437	53.42%
II	13	14.29%	78	9.54%
III	15	16.48%	174	21.27%
IV	10	10.99%	129	15.77%
X	0	0.00%	0	0.00%

### ESTIMATE and immune profile analyses

The normalized genes were utilized to evaluate the stromal, immune, and tumor purity by the ESTIMATE package in R (3.6.2). The immune infiltrations of different immune cells and immune factors for each sample were evaluated by TIMER2.0 with default parameters, including TIMER, CIBERSORT, CIBERSORT-ABS, QUANTISEQ, MCPCOUNTER, XCELL, and EPIC (http://timer.comp-genomics.org/).

### Gene ontology and Kyoto encyclopedia of genes and genomes pathway enrichment analyses

Gene Set Enrichment Analysis (GSEA) was then used for Gene Ontology (GO) and Kyoto Encyclopedia of Genes and Genomes (KEGG) analyses in R (3.6.2). The related parameter was set as follows: baseMean of ≥200, |log2 fold change (FC)| of ≥1, adj *p* value of<0.05, and minGSSize = 10. GO analyses consist of three main components: biological processes (BPs), cellular components (CCs), and molecular functions (MFs).

### Identification of prognosis-related FI-DEGs

Univariate Cox regression analyses were used to identify prognosis-related ferroptosis- and immune-related differentially expressed genes (FI-DEGs). The overlap prognostic-related FI-DEGs verified from training and validation were used to filter the independent prognostic biomarkers as measured by multivariate Cox regression analyses. In addition, risk scores were evaluated by the following formula:


$$\text{Risk score}=\sum i_{=1}^{n}(\text{Exp}i*\text{Coe}i)$$

N, Exp*i*, and Coe*i* represented gene number, level of gene expression, and coefficient value, respectively (19, 20). The Youden index from the training data was set as the cutoff value to divide the patients.

### Statistical analyses

Principal component analyses (PCA) in R 3.6.2 was used to visualize the patients with kidney cancer with different risk values in the training group, validation group, and entire group.

A repeated measures ANOVA followed by an unpaired two-tailed Student’s *t*-test was used as indicated. All results are expressed as mean ± SEM.

## Results

### Aberrant stromal statuses for KC

**Figure 1:**
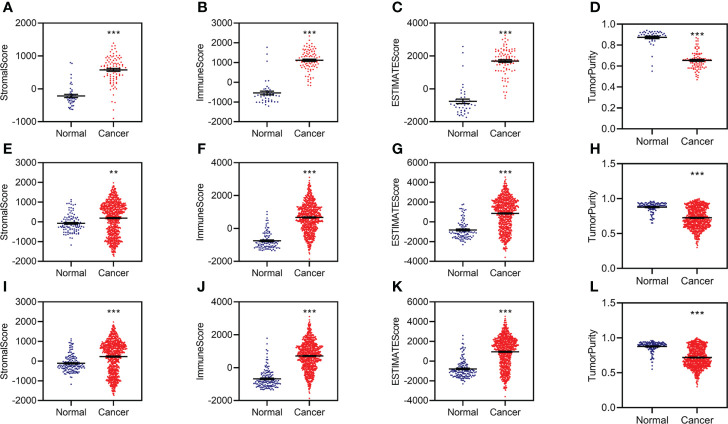
Aberrant stromal and immune status for KC. **(A–D)** Difference analysis of stromal score **(A)**, immune score **(B)**, ESTIMATE score **(C)**, and tumor purity **(D)** between normal and KC patients in the training group. **(E–H)** Difference analysis of stromal score **(E)**, immune score **(F)**, ESTIMATE score **(G)**, and tumor purity **(H)** between normal and KC patients in the validation group. **(I–L)** Difference analysis of stromal score **(I)**, immune score **(J)**, ESTIMATE score **(K)**, and tumor purity **(L)** between normal and KC patients in the entire group. **p < 0.01, ***p < 0.001.

The RNA sequencing (RNA-seq) count data were normalized by DESeq2 and used to calculate the stromal, immune, and tumor purity by ESTIMATE in R (3.6.2). The stromal, immune, and estimated scores were significantly increased, while tumor purity was significantly decreased in KC patients from the training data (**Figures 1A–D**). The estimated scores were positively correlated with stromal and immune scores and negatively correlated with tumor purity (**Supplementary Figure S1A**).

We then conducted similar analyses for KC from the validation group and found similar results in the validation group (**Figures 1E–H**; **Supplementary Figure S1B**) and the entire group (**Figures 1I–L**; **Supplementary Figure S1C**). All of these data indicated that patients with KC might have abnormal immunity.

### Identifying prognosis-related ferroptosis- and immune-related differentially expressed genes

**Figure 2:**
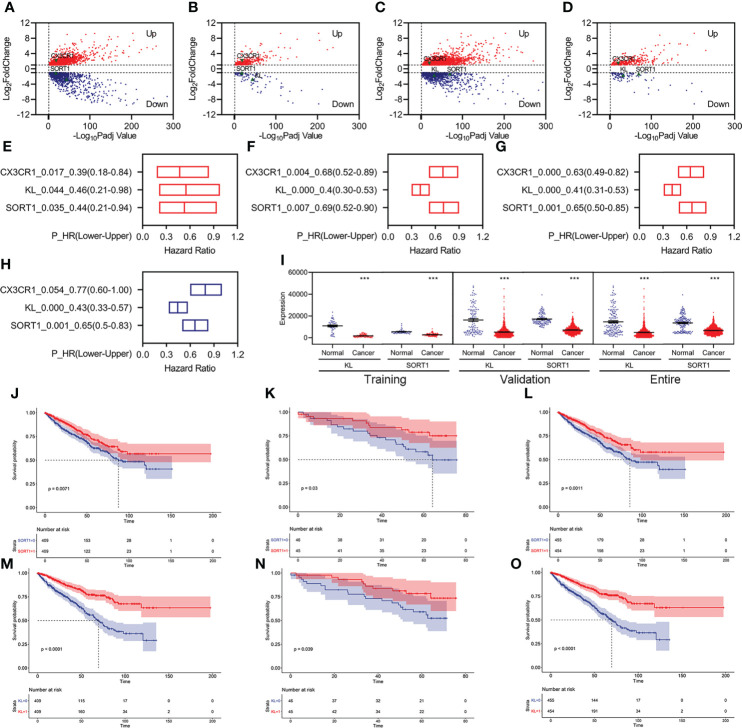
Identification of prognosis-related FI-DEGs. **(A, B)** Volcano plots of DEGs **(A)** and FI-DEGs **(B)** for KC in the training group. **(C, D)** Volcano plots of DEGs **(C)** and FI-DEGs **(D)** for KC in the validation group. **(E–G)** OS-related FI-DEGs identified by univariate Cox regression analyses in the training group **(E)**, validation group **(F)**, and entire group **(G)**. **(H)** OS-related FI-DEGs identified by multivariate Cox regression analyses in the entire group. **(I)** Expressions of KL and SORT1 in the training, validation, and entire groups. **(J–L)** KM curve of SORT1 in the training group **(J)**, validation group **(K)**, and entire group **(L)**. **(M–O)** KM curve of KL in the training group **(M)**, validation group **(N)**, and entire group **(O)**. ***p < 0.001.

We identified 3,126 differentially expressed genes (DEGs), including 1,795 upregulated and 1,331 downregulated DEGs, in the training group (**Figure 2A**). Of these, 403 FI-DEGs were identified, including 298 upregulated and 104 downregulated FI-DEGs (**Figure 2B**). We first performed univariate Cox regression analyses to obtain prognosis-related FI-DEGs and found that 33 FI-DEGs (25 upregulated and eight downregulated) were significantly correlated with KC overall survival (OS) from the training (**Supplementary Table S1**). We obtained 1,875 upregulated DEGs, 952 downregulated DEGs, 334 upregulated FI-DEGs, and 98 downregulated FI-DEGs from the validation group (**Figures 2C, D**). Univariate Cox regression analyses showed that 234 of these FI-DEGs (188 upregulated and 45 downregulated) were significantly correlated with the OS of KC (**Supplementary Table S2**).

Only three FI-DEGs (*C-X3-C Motif Chemokine Receptor 1* (*CX3CR1*), *Klotho* (*KL*), and *Sortilin 1* (*SORT1*)) were correlated with KC OS in the training and validation data (**Figures 2E, F**). Correlation analyses indicated that *KL* was negatively correlated with the immune score (**Supplementary Figure S2**). *SORT1* was negatively correlated with immune and ESTIMATE scores and positively correlated with tumor purity (**Supplementary Figure S2**). *CX3CR1* was positively correlated with immune and ESTIMATE scores and negatively correlated with tumor purity (**Supplementary Figure S2**).

We then performed Cox regression analyses for these three FI-DEGs in the entire group (ICGC and TCGA merged group) and found that they (*KL*, *SORT1*, and *CX3CR1*) were still correlated with KC OS (**Figure 2G**). Multivariate Cox regression analyses indicated that *KL* and *SORT1* were independently correlated with OS in KC (**Figure 2H**). *KL* and *SORT1* expressions were significantly decreased in the training, validation, and entire groups (**Figure 2I**). **Figures 2J–O** display the *KL* and *SORT1* Kaplan–Meier (KM) curves.

### Developing and validating the prognostic model

**Figure 3:**
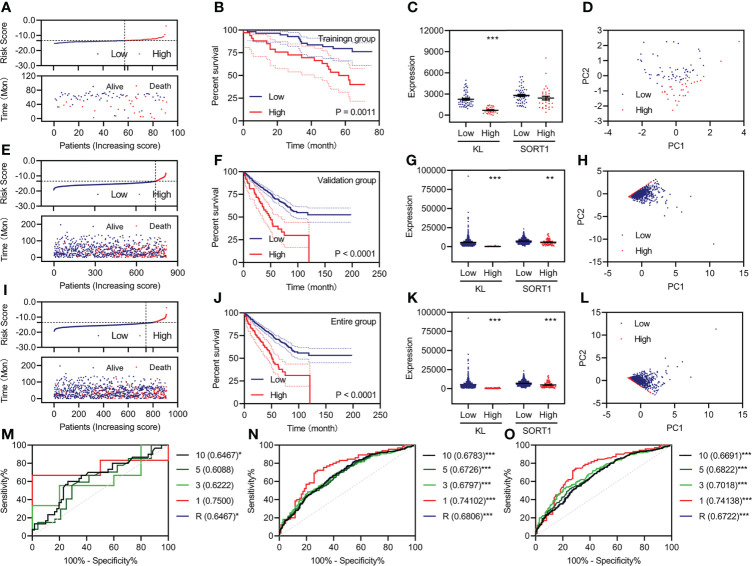
Development and validation of the prognostic model. **(A, E, I)** Risk score (up) and status (down) for each KC patient in the training group **(A)**, validation group **(E)**, and entire group **(I)**. **(B, F, J)** KM curve of a prognostic model in the training group **(B)**, validation group **(F)**, and entire group **(J)**. **(C, G, K)** Expression of KL and SORT1 among patients in the training group **(C)**, validation group **(G)**, and entire group **(K)** with different risk values. **(D, H, L)** Distribution of KC patients in the training group **(D)**, validation group **(H)**, and entire group **(L)** with different risk values. **(M–O)** Time-dependent ROC curve of a prognostic model in the training group **(M)**, validation group **(N)**, and entire group **(O)**. **p < 0.01; ***p < 0.001.

Based on previous studies, we constructed a prognostic model using two FI-DEGs (*KL* and *SORT1*). The Youden index from the training group was set as the cutoff value (**Supplementary Figure S3**). All 91 patients with KC were divided into high-risk (*n* = 34) and low-risk (*n* = 57) groups depending on the cutoff value. **Figure 3A** shows the risk score (top) and survival status (bottom) for each patient in the training group. Patients with high-risk KC had a worse OS (**Figure 3B**). *KL* expression was significantly decreased in patients with high-risk KC (**Figure 3C**). Principal component analysis (PCA) revealed that low-risk KC patients could be distinguished from high-risk KC patients (**Figure 3D**).

All 818 patients with KC in the validation group were divided into high-risk (*n* = 76) and low-risk groups (*n* = 742), depending on the cutoff value. **Figure 3E** shows each patient’s risk score (top) and survival status (bottom). Patients with high-risk KC had a worse OS (**Figure 3F**). *KL* and *SORT1* expression were significantly decreased in high-risk KC patients (**Figure 3G**). PCA revealed that low-risk KC patients could be distinguished from high-risk KC patients (**Figure 3H**). We performed similar analyses for the entire group, and **Figures 3I–L**, display the results.

Additionally, we plotted time-dependent curves for the training, validation, and entire groups. The area under the curve (AUC) of the receiver operating characteristic (ROC) curve exceeded 0.60 (**Figures 3M–O**). The values of the prognostic models at one year were over 0.70 (**Figures 3M–O**).

### Evaluating the prognostic model’s feasibility and different clinical characteristics

**Figure 4:**
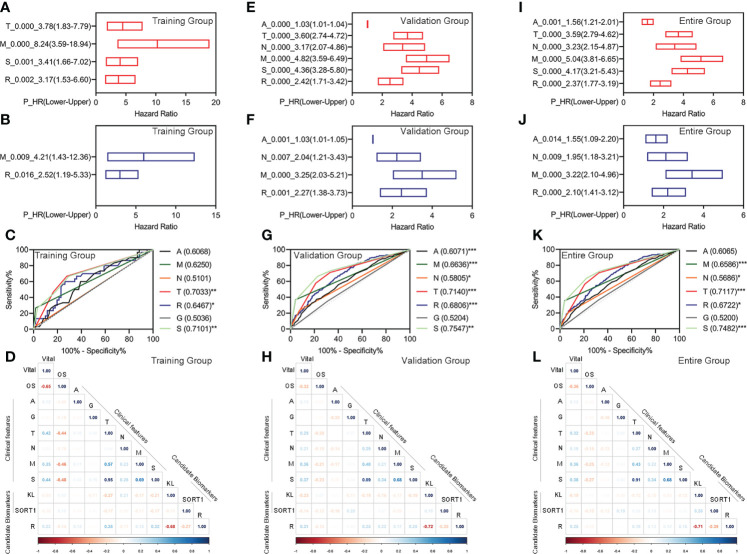
Evaluation of the feasibility of the prognostic model and different clinical characteristics. **(A–D)** Univariate Cox regression analyses **(A)**, Multivariate Cox regression analyses **(B)**, ROC curve **(C)**, and correlation analyses **(D)** for the prognostic model in the training group. **(E–H)** Univariate Cox regression analyses **(E)**, multivariate Cox regression analyses **(F)**, ROC curve **(G)**, and correlation analyses **(H)** for the prognostic model in the training group. **(I–L)** Univariate Cox regression analyses **(I)**, multivariate Cox regression analyses **(J)**, ROC curve **(K)**, and correlation analyses **(L)** for the prognostic model in the training group. *p < 0.05 **p < 0.01, ***p < 0.001.

We performed univariate Cox regression for the different clinical features and prognostic model, followed by multivariate Cox regression analyses to determine whether the prognostic model was an independent prognostic factor for KC. In the training group, we found that pathologic TM, pathologic stage, and the prognostic model were correlated with OS, as measured by univariate Cox regression (**Figure 4A**). The pathologic M and prognostic models were independently correlated with OS, as measured by multivariate Cox regression (**Figure 4B**). The ROC curve showed that the AUC value of the prognostic model was slightly higher than that of the pathologic M (**Figure 4C**). In the validation group, we found that age, pathologic TNM, pathologic stage, and the prognostic model were correlated with OS, as measured by univariate Cox regression (**Figure 4E**). Age, pathologic NM, and the prognostic model were independently correlated with OS, as measured by multivariate Cox regression (**Figure 4F**). The prognostic model had the highest AUC values among age, pathologic NM, and the prognostic model (**Figure 4G**). Similar results were obtained for all the groups (**Figures 4I–K**).

Additionally, we performed correlation analyses for different clinical features, candidate biomarkers, and prognostic models. The prognostic model significantly correlated with the candidate *KL* biomarker in the training, validation, and entire groups (**Figures 4D, H, L**).

### Investigating immune infiltration landscapes

**Figure 5:**
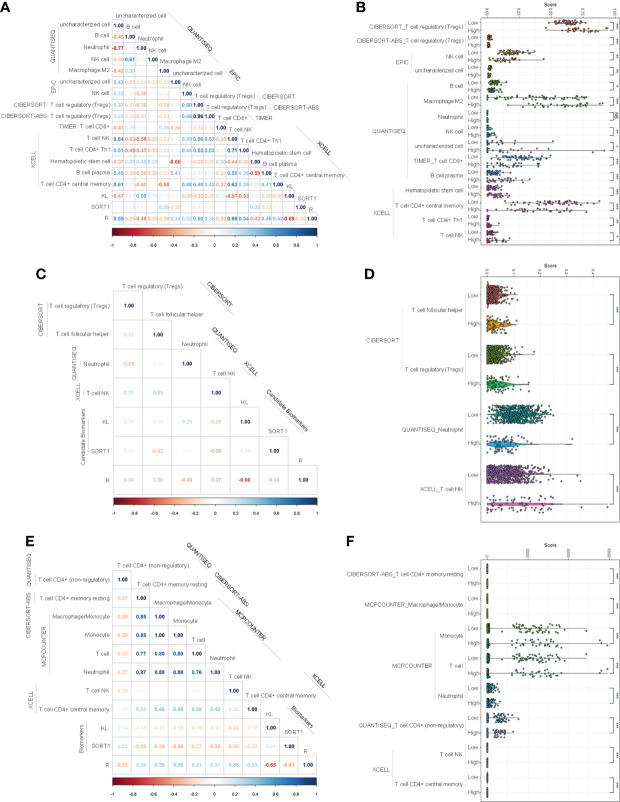
Investigation of immune infiltration landscapes. **(A, B)** Correlation analyses **(A)** and difference analyses **(B)** of several immune cells and factors with significant correlation with the prognostic model in the training group. **(C, D)** Correlation analyses **(C)** and difference analyses **(D)** of several immune cells and factors with significant correlation with the prognostic model in validation group. **(E, F)** Correlation analyses **(E)** and difference analyses **(F)** of several immune cells and factors with significant correlation with the prognostic model of the entire group. *p < 0.05; **p < 0.01; ***p < 0.001. NS means No Significance.

We used the normalized expression data of genes to evaluate the immune infiltration of immune cells and factors using TIMER2.0 and determine the immune infiltration landscapes of KC patients. In the training group, 85 immune cells and factors significantly differed between normal and KC patients (**Supplementary Table S3**). Of these, 27 immune cells and factors significantly differed between KC patients with high-risk and low-risk values. We performed correlation analyses to clarify which immune cells and factors were associated with the prognostic model and found that the prognostic model was significantly correlated with 15 immune cells and factors, such as NK cells, T-cell CD8+, T-cell regulatory, etc. (**Figure 5A**). **Figure 5B** displays the expression of these 15 immune cells and factors.

In the validation group, 92 immune cells and factors significantly differed between the normal and KC patients. Of these, 49 significantly differed between KC patients with high-risk values and KC patients with low-risk values. Correlation analyses indicated that the prognostic model was significantly correlated with four immune cells and factors, such as T-cell NK, T-cell regulatory, etc. (**Figure 5C**). **Figure 5D** displays the expression of these 15 immune cells and factors.

In the entire group, 92 immune cells and factors significantly differed between the normal and cancer groups (**Supplementary Table S5**). Of these, 49 significantly differed among the KC patients with different risk values. Correlation analyses indicated that eight immune cells and factors were significantly correlated with the prognostic model, such as T-cell NK, T cells, and macrophages/monocytes (**Figure 5E**). **Figure 5F** displays the expression of these eight immune cells and factors.

### GO and KEGG pathway enrichment analyses

**Figure 6:**
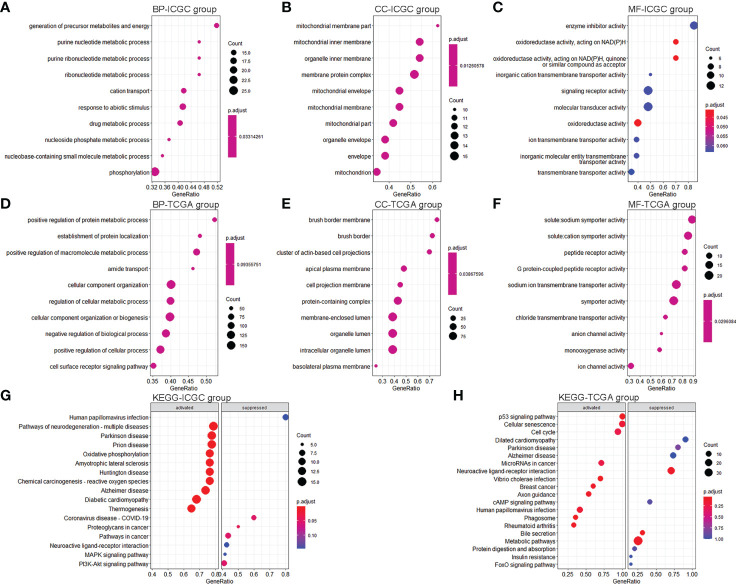
GO and KEGG pathway enrichment analyses. **(A–C)** Top 10 enrichment BP **(A)**, CC **(B)**, and MF **(C)** between KC patients with a high-risk value and low-risk value in the training group. **(D–F)** Top 10 enrichment BP **(D)**, CC **(E)**, and MF **(F)** between KC patients with a high-risk value and low-risk value in the validation group. **(G)** Top 10 enrichment KEGG between KC patients with a high-risk value and low-risk value in the training group. **(H)** Top 10 enrichment KEGG between KC patients with a high-risk value and low-risk value in the validation group.

We conducted differential expression analyses to understand the differences in ferroptosis- and immune-related pathways between high-risk and low-risk KC. We obtained 542 DEGs (476 upregulated and 66 downregulated) from the training data (**Supplementary Figure S4A**) and 1,169 DEGs (669 upregulated and 500 downregulated) from the validation data (**Supplementary Figure S4B**). GSEA was then used for GO and KEGG analyses. We found that 132 BP, 39 MF, and 17 CC were significantly enriched in the training group (**Supplementary Table S6**). There were several ferroptosis-related GO terms, such as oxidative phosphorylation (BP) and oxidoreductase activity (MF) (**Supplementary Table S6**). KEGG analysis indicated that 19 signaling pathways were enriched (**Supplementary Table S7**). Of these, 16 were significantly enriched, including several ferroptosis- and immune-related pathways, such as oxidative phosphorylation, chemical carcinogenesis, reactive oxygen species (ROS), and PI3K-Akt. **Figures 6A–C, G** show the top 10 GO and KEGG signaling pathways. Furthermore, 314 BP, 65 MF, and 58 CC were significantly enriched in the validation group (**Supplementary Table S8**). There were several ferroptosis- and immune-related GO terms, such as immune response-regulating signaling pathway (BP), immune response-activating signal transduction (BP), and oxidoreductase activity (MF). KEGG analysis indicated that 77 signaling pathways were enriched (**Supplementary Table S9**). Of these, 13 were significantly enriched, including several ferroptosis- and immune-related pathways, such as the p53 signaling pathway. **Figures 6D–F, H** show the top 10 GO and KEGG signaling pathways.

## Discussion

Recently, immunotherapy and ferroptosis regulation have been identified as potential cancer therapeutic strategies (16–18). Interestingly, immune responses and ferroptosis can regulate each other to achieve their anticancer effects. Here, we conducted an integrated analysis of ferroptosis- and immune-related genes to identify suitable biomarkers to predict the prognosis and immune status of KC patients. We found that two FI-DEGs (*KL* and *SORT1*) independently correlated with the OS of KC. The prognostic model using these two FI-DEGs could predict the outcome and immune status of KC. Moreover, we found that the prognostic model was significantly correlated with several immune cells. Several ferroptosis- and immune-related GO and KEGG terms were enriched, reinforcing the role of ferroptosis and immunity in KC development (12–15).

KL is an age-suppressing protein secreted by the kidneys, brain, and thyroid gland. Previous studies have demonstrated that KL can suppress tumor growth, inhibit metastases, reduce resistance, and improve survival. For example, Doi et al. found that KL suppresses cancer metastasis and improves survival in mice transplanted with cancer cells (21). Ligumsky et al. found that KL overexpression could inhibit colony formation in MCF-7 and MDA-MB-231 cells (22). Dai et al. reported that KL inhibits cell growth and promotes apoptosis in thyroid cancer (23). KL is a potential tumor suppressor. Previous studies have also demonstrated that KL is downregulated in several cancers, such as pancreatic cancer and hepatocellular carcinoma (HCC) (24). KL could be a prognostic biomarker for several cancers, such as ovarian cancer and head and neck squamous carcinomas (25, 26). In the present study, we found that KL expression was significantly decreased in KC patients, KC patients with high-risk values, and KC patients with metastasis. These results reinforced KL’s role as a tumor suppressor. Patients with low KL expression had a worse OS. This result is consistent with a previous report’s trend that KL overexpression can prolong survival time (21). Most importantly, Zhu et al. confirmed that KL suppresses tumor progression by inhibiting PI3K/Akt/GSK3β/Snail signaling in RCC (27).

SORT1 is a lysosomal trafficking receptor. Liang et al. found that SORT1 upregulation promotes gastric cancer progression. Previous studies have shown that SORT1 is associated with drug resistance. Yamamoto et al. found that suppressing SORT1 in lenalidomide-resistant cells restored drug sensitivity (28). Charfi et al. detected the SORT1 receptor in 3D capillary-like structures formed by ES-2 ovarian cancer cells and MDA-MB-231 TNBC-derived cells *in vitro*. SORT1 suppression inhibits capillary-like structure formation. SORT1 overexpression is associated with poor prognosis in colorectal cancer (29). In the present study, KC patients displayed significantly decreased SORT1 expression. Patients with low KL expression had a worse OS. These results indicate that SORT1 may play different roles in different cancers.

Previous studies have demonstrated that *KL* and *SORT1* are correlated with immunity and ferroptosis. For example, Lai et al. found that a KL deficiency significantly increased the proportion of cluster of differentiation (CD)68+/CD11b+ cells (the source of mononuclear macrophage M1 cells) in peripheral blood (30). Mytych et al. found that *KL* decreased ROS/reactive nitrogen species (RNS) and pro-inflammatory cytokine levels in lipopolysaccharide (LPS)-treated monocytes and upregulated anti-inflammatory interleukin (IL)-10 secretion (31). Sato et al. indicated that acquired immune responses were hardly induced in *KL* knockout mice (32). Murine and human macrophages and dendritic cells, which are crucial in innate immunity but not adaptive immunity, profoundly express SORT1 (33). Mortensen et al. found that SORT1 is a high-affinity receptor for pro-inflammatory cytokines IL-6 and interferon-gamma (IFN-γ) (34). The present study found that KL expression was significantly correlated with several immune cells, reinforcing the relationship between *KL*, *SORT1*, and immunity.

Using the same methods and parameters, we found that *KL* and *SORT1* could be prognostic biomarkers for KC. However, Human Pathology Atlas data indicated that KL is a prognostic biomarker for KC, while SORT1 has low specificity. Future studies should verify whether *SORT1* could be used as a KC biomarker. Although our prognostic model based on *KL* and *SORT1* can better predict the prognosis of KC, its clinical use requires further study.

## Conclusion

Comprehensive analyses indicated that two FI-DEGs (*KL* and *SORT1*) were independently correlated with the OS of KC patients. Prognostic models using these two FI-DEGs could accurately predict KC patient outcomes and immune landscapes. Although we constructed and validated the risk model with two independent samples, further research is needed to determine whether it could be used clinically and how these molecules are involved in KC mechanisms.

## Data availability statement

The original contributions presented in the study are included in the article/**Supplementary Material**. Further inquiries can be directed to the corresponding author.

## Author contributions

X-LX and ZH: conceived and designed the experiments. X-LX, ZH, and HW: performed the analyses. YQ and JL: helped to analyze the data. X-LX: wrote the paper. All authors listed have made a substantial, direct, and intellectual contribution to the work and approved it for publication.

## Funding

This project is financially supported by the Doctor Foundation of Hunan University of medicine (2020122004), Hunan Provincial Education Department (20B417).

## Conflict of interest

The authors declare that the research was conducted in the absence of any commercial or financial relationships that could be construed as a potential conflict of interest.

## Publisher’s note

All claims expressed in this article are solely those of the authors and do not necessarily represent those of their affiliated organizations, or those of the publisher, the editors and the reviewers. Any product that may be evaluated in this article, or claim that may be made by its manufacturer, is not guaranteed or endorsed by the publisher.
