# Supplementary material for: Establishing a prognostic model of ferroptosis- and immune-related signatures in kidney cancer: A study based on TCGA and ICGC databases
Source: Front Oncol. 2022 Aug 26;12:931383. doi: 10.3389/fonc.2022.931383 (PMC9459019; doi:10.3389/fonc.2022.931383)
Supplement: Supplementary file 1 [file DataSheet_1.docx]

**for ESTIMATE**

library(estimate)

data<-read.table('C:/Users/1/Desktop/1/XXL_group.txt',row.names = 1,header = T,sep="\t")

data[1:4,1:4]

filterCommonGenes(input.f = 'C:/Users/1/Desktop/1/XXL_group.txt',output.f = 'C:/Users/1/Desktop/1/XXL_group1.gct',id='GeneSymbol')

estimateScore('C:/Users/1/Desktop/1/XXL_group1.gct','C:/Users/1/Desktop/1/XXL_purity1.gct')

object<-read.table('C:/Users/1/Desktop/1/XXL_purity1.gct',skip = 2,header = T)

write.csv(object,file="xxl.csv")

**for DEGs**

XXL_count<-read.table("XXL_RAW_Counts.txt",sep="\t",header=TRUE)

rownames(XXL_count)<-XXL_count[,1]

XXL_count<-XXL_count[,2:520]

XXL_coldata<-read.table("XXL_coldata.txt",sep="\t",header=TRUE)

rownames(XXL_coldata)<-XXL_coldata[,1]

View(XXL_coldata)

library("DESeq2")

colnames(XXL_count)<-gsub("[:.:]","-",colnames(XXL_count))

ddsXXL<-DESeqDataSetFromMatrix(countData = XXL_count,colData = XXL_coldata, design = ~condition)

ddsXXL_collapsed<-collapseReplicates(ddsXXL,groupby = ddsXXL$sample,run=ddsXXL$run)

ddsXXL_collapsed$condition<-relevel(ddsXXL_collapsed$condition,"Normal")

as.data.frame(colData(ddsXXL_collapsed))

keep<-rowSums(counts(ddsXXL_collapsed))>=100

ddsXXL_collapsed<-DESeq(ddsXXL_collapsed,parallel = TRUE)

XXL_res<-results(ddsXXL_collapsed)

summary(XXL_res)

write.csv(as.data.frame(XXL_res),file="XXL_DEGenes.csv")

write.csv(as.data.frame(counts(ddsXXL_collapsed,normalized=TRUE)),file="XXL_normalized_table.csv")

**for Univariate Cox regression analyses**

library(survival)

library(survminer)

library(RegParallel)

res5 <- RegParallel(data = xxl, formula = 'Surv(os, vital) ~ [*]', FUN = function(formula, data) coxph(formula = formula, data = data, ties = 'breslow', singular.ok = TRUE), FUNtype = 'coxph', variables = colnames(xxl)[4:ncol(xxl)], blocksize = 10, p.adjust = "BH")

write.csv(res5,file="xxl.csv")

**for Multivariate Cox regression analyses**

library(survival)

cox2 <- coxph(Surv(os, vital) ~ ENSG00000262001+ENSG00000234912+ENSG00000275437+ENSG00000206573+ENSG00000172965+ENSG00000273142+ENSG00000232442+ENSG00000242125+ENSG00000234072+ENSG00000170919+ENSG00000130600+ENSG00000273344+ENSG00000263072, data = xxl)

cox2

summary(cox2)

**for PCA**

x<-as.matrix(XXL[,10:ncol(XXL)])

rownames(x)<-XXL$case_submitter_id

PCA<-prcomp(x,center = T,scale=T )

write.csv(PCA$x,file="PCA.csv")
