# Supplementary material for: Establishing a prognostic model of ferroptosis- and immune-related signatures in kidney cancer: A study based on TCGA and ICGC databases
Source: Front Oncol. 2022 Aug 26;12:931383. doi: 10.3389/fonc.2022.931383 (PMC9459019; doi:10.3389/fonc.2022.931383)
Supplement: Supplementary file 2 [file DataSheet_2.docx]

**Establishing a prognostic model of ferroptosis- and immune-related signatures in kidney cancer: A study based on TCGA and ICGC databases**

Zhijun Han^1#^, Hao Wang^3#,^ Jing Long^1^, Yanning Qiu^4^, Xiao-Liang Xing^2,3*^

^1^Department of Urology, Department of Ultrasonography, Zhuzhou Hospital Affiliated to Xiangya school of Medicine, Central South University, Zhuzhou 412000, Hunan, P. R. China.

^2^Hunan Provincial Key Laboratory for Synthetic Biology of Traditional Chinese Medicine, Hunan University of Medicine, Huaihua 418000, Hunan, P. R. China.

^3^Department of Urology, The First Affiliated Hospital to Hengyang Medical School, South China University, Hengyang 421000, Hunan, P. R. China.

^4^First College for Clinical Medicine, Xinjiang Medical University, Urumqi 830000, P. R. China.

**^#^contributed equally to this work**

**^*^correspondence author:** Xiao-Liang Xing, xiaoliangxinghnm@126.com

# Supplementary: 4 figures and 9 tables


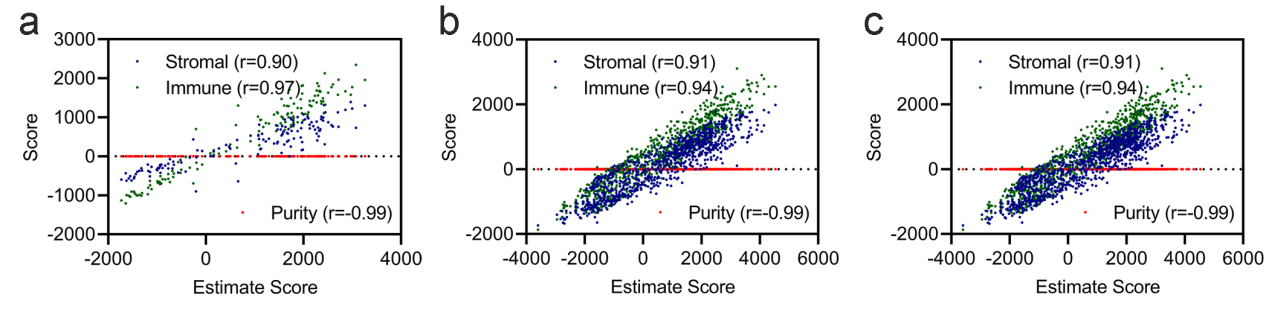


# Supplementary Figure 1 Correlation analyses for ESTIMATE score with the stromal score, immune score, and tumor purity.

a, correlation analyses in training group. b, correlation analyses in validation group. c, correlation analyses in entire group.


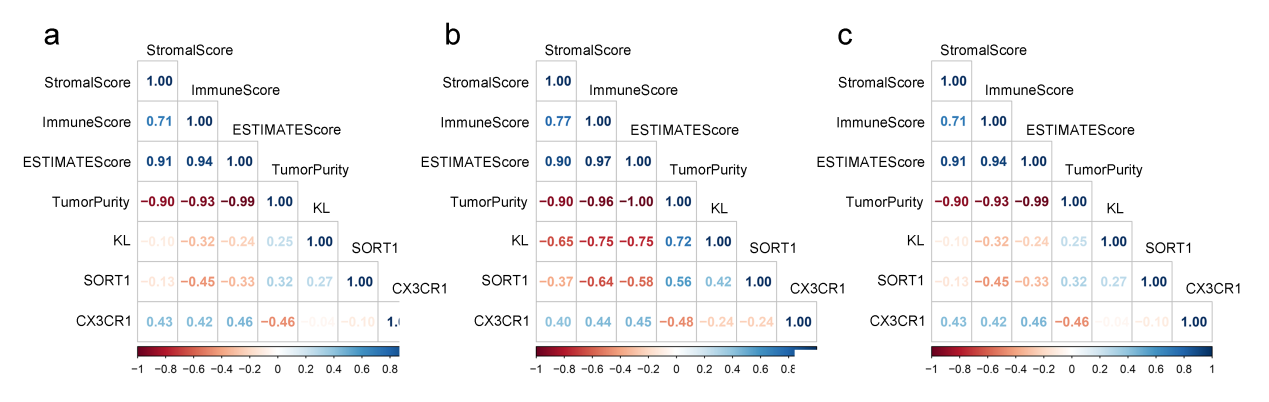


# Supplementary Figure 2 Correlation analyses for KL, SORT1, and CX3CR1 with the Estimate score.

a, correlation analyses in training group. b, correlation analyses in validation group. c, correlation analyses in entire group.


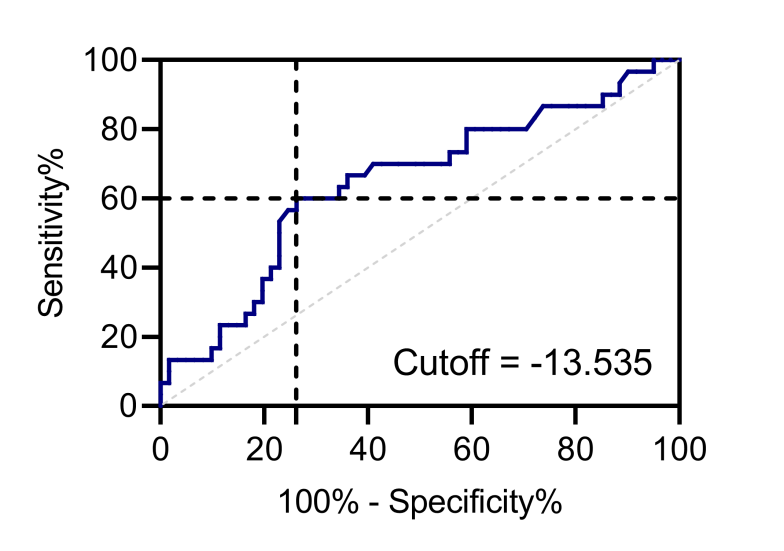


# Supplementary Figure 3 Yonden index from training group was set as the optimal cutoff value to divide KC patients.

**
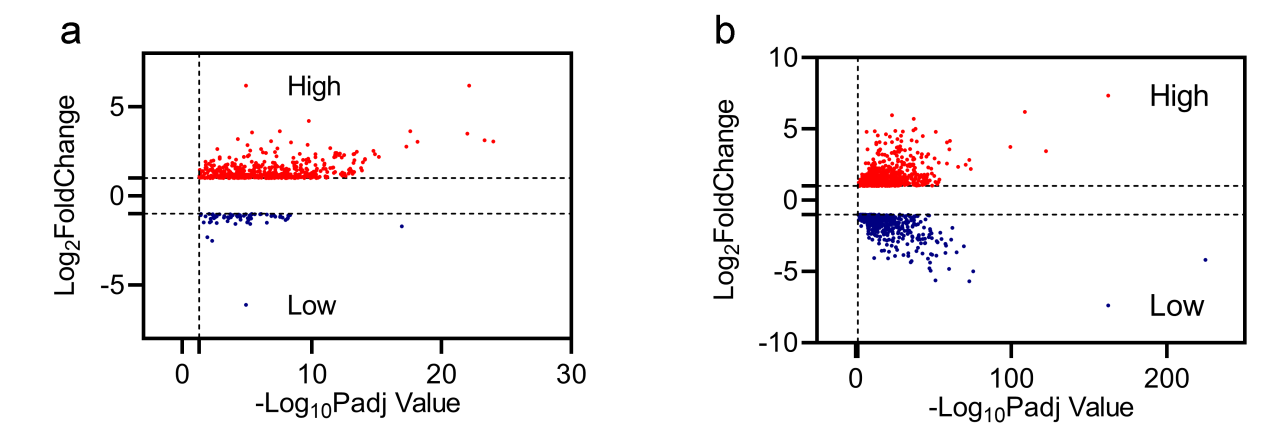
**

# Supplementary Figure 4 Differential expression analyses for KC patients with different risk value

a, Volcano plot of DEGs for KC in training group. b, Volcano plot of DEGs for KC in validation group.

# Supplementary Table 1 OS related FI-DEGs identified by univariate Cox regression analyses for the KC patients in training group.

| Symbol | | P | HR | HRlower | HRupper |
| --- | --- | --- | --- | --- | --- |
| upregulated | CXCR4 | 0.016 | 0.39 | 0.18 | 0.84 |
|  | NGFR | 0.004 | 0.30 | 0.13 | 0.69 |
|  | PLXND1 | 0.031 | 0.43 | 0.20 | 0.93 |
|  | LCP2 | 0.007 | 0.33 | 0.15 | 0.74 |
|  | CX3CR1 | 0.017 | 0.39 | 0.18 | 0.84 |
|  | SEMA3F | 0.034 | 0.44 | 0.20 | 0.94 |
|  | NFATC2 | 0.017 | 0.38 | 0.18 | 0.84 |
|  | CD4 | 0.021 | 0.40 | 0.18 | 0.87 |
|  | EDNRA | 0.011 | 0.36 | 0.17 | 0.80 |
|  | SDC3 | 0.022 | 0.40 | 0.18 | 0.87 |
|  | FLT4 | 0.028 | 0.42 | 0.20 | 0.91 |
|  | PDGFRB | 0.010 | 0.36 | 0.16 | 0.78 |
|  | STING1 | 0.019 | 0.39 | 0.18 | 0.85 |
|  | TNFRSF14 | 0.008 | 2.90 | 1.33 | 6.34 |
|  | JAG1 | 0.025 | 0.42 | 0.20 | 0.90 |
|  | PLXNA2 | 0.008 | 0.35 | 0.16 | 0.76 |
|  | CETP | 0.049 | 0.47 | 0.22 | 1.00 |
|  | NRP1 | 0.003 | 0.29 | 0.13 | 0.65 |
|  | PDGFB | 0.038 | 0.45 | 0.21 | 0.96 |
|  | MX2 | 0.020 | 0.41 | 0.19 | 0.87 |
|  | CSF2RB | 0.033 | 0.44 | 0.20 | 0.94 |
|  | F2R | 0.037 | 0.45 | 0.21 | 0.95 |
|  | IFNAR2 | 0.019 | 0.39 | 0.18 | 0.86 |
|  | APLNR | 0.038 | 0.45 | 0.21 | 0.96 |
|  | RARG | 0.033 | 0.44 | 0.20 | 0.93 |
| downregulated | SORT1 | 0.035 | 0.44 | 0.21 | 0.94 |
|  | ESR1 | 0.045 | 0.46 | 0.22 | 0.98 |
|  | SEMA5A | 0.021 | 0.40 | 0.18 | 0.87 |
|  | CCL21 | 0.027 | 0.42 | 0.20 | 0.91 |
|  | VAV3 | 0.049 | 2.12 | 1.00 | 4.48 |
|  | PLCG2 | 0.030 | 0.43 | 0.20 | 0.92 |
|  | S100A14 | 0.049 | 2.09 | 1.00 | 4.36 |
|  | KL | 0.044 | 0.46 | 0.21 | 0.98 |

# Supplementary Table 2 OS related FI-DEGs identified by univariate Cox regression analyses for the KC patients in validation group.

| Symbol | | P | HR | HRlower | HRupper |
| --- | --- | --- | --- | --- | --- |
| upregulated | FABP6 | 0.000 | 1.85 | 1.39 | 2.46 |
|  | CD70 | 0.010 | 1.43 | 1.09 | 1.88 |
|  | CHIT1 | 0.028 | 0.74 | 0.57 | 0.97 |
|  | IL20RB | 0.000 | 3.31 | 2.42 | 4.53 |
|  | PTHLH | 0.000 | 1.69 | 1.28 | 2.24 |
|  | MCHR1 | 0.008 | 1.44 | 1.10 | 1.90 |
|  | ALOX15B | 0.031 | 0.74 | 0.57 | 0.97 |
|  | CDKN2A | 0.001 | 1.60 | 1.22 | 2.11 |
|  | PGF | 0.005 | 1.50 | 1.13 | 1.98 |
|  | SAA1 | 0.000 | 2.03 | 1.53 | 2.68 |
|  | SST | 0.046 | 0.76 | 0.58 | 0.99 |
|  | SAA2 | 0.000 | 2.10 | 1.59 | 2.77 |
|  | EPO | 0.043 | 1.32 | 1.01 | 1.73 |
|  | NNMT | 0.004 | 1.50 | 1.14 | 1.97 |
|  | SCG2 | 0.000 | 1.80 | 1.36 | 2.38 |
|  | IDO1 | 0.017 | 1.40 | 1.06 | 1.85 |
|  | TNFRSF9 | 0.000 | 1.70 | 1.29 | 2.24 |
|  | TNFSF14 | 0.000 | 2.13 | 1.60 | 2.85 |
|  | CXCL13 | 0.000 | 1.92 | 1.45 | 2.56 |
|  | INHBB | 0.046 | 1.32 | 1.01 | 1.74 |
|  | MMP9 | 0.009 | 1.43 | 1.09 | 1.88 |
|  | CXCL11 | 0.011 | 1.43 | 1.08 | 1.88 |
|  | CD8A | 0.006 | 1.47 | 1.12 | 1.94 |
|  | CCL20 | 0.000 | 1.73 | 1.31 | 2.29 |
|  | FCGR3A | 0.002 | 1.55 | 1.17 | 2.04 |
|  | CXCL9 | 0.027 | 1.36 | 1.04 | 1.78 |
|  | AQP9 | 0.000 | 1.80 | 1.36 | 2.37 |
|  | TF | 0.000 | 2.14 | 1.61 | 2.83 |
|  | CXCL10 | 0.023 | 1.37 | 1.05 | 1.80 |
|  | TRIB3 | 0.000 | 2.31 | 1.73 | 3.10 |
|  | CCL5 | 0.001 | 1.58 | 1.20 | 2.09 |
|  | PDCD1 | 0.000 | 1.67 | 1.27 | 2.20 |
|  | SLC7A11 | 0.000 | 1.68 | 1.27 | 2.21 |
|  | TYMP | 0.007 | 1.45 | 1.11 | 1.91 |
|  | TNFRSF4 | 0.033 | 1.35 | 1.02 | 1.78 |
|  | FGA | 0.020 | 1.38 | 1.05 | 1.81 |
|  | GNLY | 0.001 | 1.63 | 1.24 | 2.15 |
|  | BIRC5 | 0.000 | 2.58 | 1.92 | 3.46 |
|  | CCR5 | 0.047 | 1.32 | 1.00 | 1.73 |
|  | CD3D | 0.002 | 1.56 | 1.18 | 2.06 |
|  | TRBV28 | 0.000 | 1.68 | 1.27 | 2.21 |
|  | IL2RB | 0.000 | 1.69 | 1.28 | 2.23 |
|  | FCER1G | 0.017 | 1.39 | 1.06 | 1.82 |
|  | CXCR3 | 0.010 | 1.43 | 1.09 | 1.88 |
|  | CXCR4 | 0.000 | 1.70 | 1.29 | 2.24 |
|  | TRBC2 | 0.000 | 1.66 | 1.26 | 2.20 |
|  | CD72 | 0.000 | 1.74 | 1.32 | 2.30 |
|  | LBP | 0.000 | 1.79 | 1.36 | 2.36 |
|  | CCL4 | 0.048 | 1.31 | 1.00 | 1.72 |
|  | CXCR6 | 0.007 | 1.45 | 1.11 | 1.90 |
|  | ZAP70 | 0.000 | 1.75 | 1.33 | 2.31 |
|  | VIM | 0.001 | 1.60 | 1.21 | 2.11 |
|  | SLC11A1 | 0.001 | 1.62 | 1.23 | 2.12 |
|  | TMSB10 | 0.016 | 1.39 | 1.06 | 1.82 |
|  | LTB4R | 0.000 | 2.47 | 1.85 | 3.29 |
|  | RRM2 | 0.000 | 2.48 | 1.85 | 3.32 |
|  | CD3E | 0.002 | 1.53 | 1.16 | 2.02 |
|  | PRF1 | 0.012 | 1.42 | 1.08 | 1.87 |
|  | TNFSF13B | 0.000 | 1.96 | 1.48 | 2.61 |
|  | IL12RB1 | 0.002 | 1.53 | 1.17 | 2.01 |
|  | GZMB | 0.001 | 1.62 | 1.23 | 2.15 |
|  | CD247 | 0.001 | 1.57 | 1.19 | 2.07 |
|  | ISG20 | 0.000 | 1.69 | 1.28 | 2.22 |
|  | NOD2 | 0.000 | 2.05 | 1.54 | 2.72 |
|  | NMB | 0.018 | 1.39 | 1.06 | 1.84 |
|  | EBI3 | 0.004 | 1.49 | 1.14 | 1.96 |
|  | IL21R | 0.000 | 1.99 | 1.49 | 2.65 |
|  | ITGAL | 0.007 | 1.46 | 1.11 | 1.92 |
|  | CD3G | 0.030 | 1.35 | 1.03 | 1.78 |
|  | VAV1 | 0.009 | 1.43 | 1.09 | 1.88 |
|  | CARD11 | 0.020 | 1.38 | 1.05 | 1.80 |
|  | APOBEC3C | 0.006 | 1.46 | 1.11 | 1.92 |
|  | APOBEC3G | 0.000 | 2.09 | 1.57 | 2.79 |
|  | IL10RA | 0.001 | 1.60 | 1.22 | 2.10 |
|  | MASP1 | 0.011 | 0.71 | 0.54 | 0.92 |
|  | HSPA6 | 0.002 | 1.55 | 1.18 | 2.04 |
|  | RAC2 | 0.001 | 1.56 | 1.19 | 2.05 |
|  | IGLV1-47 | 0.000 | 1.94 | 1.47 | 2.58 |
|  | IL2RA | 0.003 | 1.51 | 1.15 | 1.98 |
|  | LCP2 | 0.014 | 1.41 | 1.07 | 1.85 |
|  | IGLV1-40 | 0.001 | 1.58 | 1.20 | 2.08 |
|  | BMP1 | 0.000 | 1.91 | 1.45 | 2.51 |
|  | FABP5 | 0.000 | 1.82 | 1.38 | 2.41 |
|  | HLA-DOB | 0.004 | 1.49 | 1.13 | 1.96 |
|  | HCST | 0.000 | 2.08 | 1.57 | 2.77 |
|  | GBP2 | 0.000 | 1.98 | 1.49 | 2.63 |
|  | IGLV6-57 | 0.000 | 1.67 | 1.27 | 2.20 |
|  | MC1R | 0.008 | 1.44 | 1.10 | 1.88 |
|  | IGHA2 | 0.020 | 1.38 | 1.05 | 1.81 |
|  | DDIT4 | 0.006 | 1.46 | 1.11 | 1.92 |
|  | IGHG1 | 0.000 | 1.85 | 1.40 | 2.44 |
|  | IGLV8-61 | 0.016 | 1.39 | 1.06 | 1.83 |
|  | ITK | 0.018 | 1.39 | 1.06 | 1.82 |
|  | AGER | 0.000 | 1.62 | 1.24 | 2.13 |
|  | IGHV3-30 | 0.001 | 1.61 | 1.22 | 2.12 |
|  | ACKR3 | 0.002 | 1.57 | 1.18 | 2.09 |
|  | IGLV3-10 | 0.017 | 1.39 | 1.06 | 1.83 |
|  | IGLV3-21 | 0.001 | 1.62 | 1.23 | 2.13 |
|  | TRAC | 0.004 | 1.50 | 1.14 | 1.98 |
|  | TAP1 | 0.011 | 1.42 | 1.08 | 1.87 |
|  | IGLV3-19 | 0.000 | 1.65 | 1.25 | 2.18 |
|  | IGLC3 | 0.000 | 2.21 | 1.66 | 2.94 |
|  | IGHV1-46 | 0.006 | 1.47 | 1.12 | 1.92 |
|  | SLC2A1 | 0.007 | 1.46 | 1.11 | 1.93 |
|  | IGHG3 | 0.000 | 2.14 | 1.61 | 2.84 |
|  | IGHV3-21 | 0.000 | 1.66 | 1.26 | 2.18 |
|  | SLC2A3 | 0.001 | 1.61 | 1.22 | 2.13 |
|  | NGF | 0.045 | 1.32 | 1.01 | 1.74 |
|  | CMTM3 | 0.010 | 1.42 | 1.09 | 1.86 |
|  | TNFRSF10B | 0.036 | 1.33 | 1.02 | 1.74 |
|  | CD14 | 0.001 | 1.59 | 1.21 | 2.08 |
|  | IL2RG | 0.000 | 1.65 | 1.25 | 2.19 |
|  | IGLV3-25 | 0.000 | 1.71 | 1.29 | 2.25 |
|  | IGHV3-49 | 0.003 | 1.52 | 1.16 | 2.00 |
|  | IGHV1-24 | 0.005 | 1.48 | 1.13 | 1.95 |
|  | PDGFD | 0.000 | 0.62 | 0.47 | 0.81 |
|  | IGKV1-17 | 0.017 | 1.39 | 1.06 | 1.83 |
|  | IGKV3-11 | 0.001 | 1.62 | 1.23 | 2.13 |
|  | IGLV1-51 | 0.001 | 1.57 | 1.19 | 2.06 |
|  | IGKC | 0.000 | 1.90 | 1.44 | 2.52 |
|  | IGLV1-44 | 0.000 | 1.89 | 1.43 | 2.51 |
|  | AKR1C2 | 0.017 | 0.72 | 0.55 | 0.94 |
|  | PLAUR | 0.000 | 1.75 | 1.33 | 2.31 |
|  | ISG15 | 0.000 | 1.83 | 1.39 | 2.42 |
|  | IGHV1-18 | 0.000 | 1.66 | 1.27 | 2.19 |
|  | IGHM | 0.017 | 1.39 | 1.06 | 1.83 |
|  | IGHA1 | 0.000 | 1.77 | 1.34 | 2.33 |
|  | OASL | 0.000 | 1.74 | 1.31 | 2.30 |
|  | IGHV4-39 | 0.000 | 1.72 | 1.30 | 2.27 |
|  | IGLV4-69 | 0.001 | 1.58 | 1.20 | 2.07 |
|  | SYTL1 | 0.002 | 1.53 | 1.16 | 2.00 |
|  | CX3CR1 | 0.004 | 0.68 | 0.52 | 0.89 |
|  | CD44 | 0.006 | 1.46 | 1.11 | 1.92 |
|  | IRF9 | 0.002 | 1.53 | 1.17 | 2.01 |
|  | IRF7 | 0.000 | 1.72 | 1.31 | 2.27 |
|  | IGLV2-23 | 0.001 | 1.60 | 1.22 | 2.11 |
|  | IGKV3-15 | 0.000 | 1.72 | 1.31 | 2.27 |
|  | IGLV3-1 | 0.004 | 1.49 | 1.13 | 1.96 |
|  | IGLC2 | 0.000 | 1.90 | 1.43 | 2.51 |
|  | IL4R | 0.001 | 1.55 | 1.18 | 2.04 |
|  | IGLV2-8 | 0.008 | 1.45 | 1.10 | 1.91 |
|  | OSMR | 0.009 | 1.44 | 1.09 | 1.89 |
|  | NFKBIZ | 0.001 | 1.58 | 1.20 | 2.06 |
|  | PROCR | 0.001 | 1.61 | 1.22 | 2.12 |
|  | IGHV3-15 | 0.012 | 1.42 | 1.08 | 1.86 |
|  | TNFRSF25 | 0.040 | 1.33 | 1.01 | 1.74 |
|  | S100A8 | 0.036 | 1.34 | 1.02 | 1.75 |
|  | PLXNA3 | 0.007 | 1.45 | 1.11 | 1.90 |
|  | IGHV3-33 | 0.004 | 1.50 | 1.14 | 1.97 |
|  | IGKV3-20 | 0.000 | 1.67 | 1.27 | 2.21 |
|  | IGKV1-6 | 0.007 | 1.45 | 1.10 | 1.90 |
|  | IGHV5-51 | 0.000 | 1.75 | 1.33 | 2.31 |
|  | IGKV1-16 | 0.003 | 1.52 | 1.16 | 2.00 |
|  | IGHV3-11 | 0.005 | 1.48 | 1.13 | 1.95 |
|  | IGKV4-1 | 0.000 | 1.63 | 1.24 | 2.15 |
|  | TGFB1 | 0.000 | 1.95 | 1.46 | 2.58 |
|  | STEAP3 | 0.000 | 2.48 | 1.86 | 3.30 |
|  | LMBR1L | 0.000 | 1.65 | 1.26 | 2.16 |
|  | IGHV3-23 | 0.000 | 1.69 | 1.28 | 2.22 |
|  | IGHV4-59 | 0.000 | 1.83 | 1.39 | 2.42 |
|  | CKLF | 0.000 | 1.73 | 1.31 | 2.27 |
|  | TNFRSF1B | 0.025 | 1.36 | 1.04 | 1.79 |
|  | CD28 | 0.016 | 1.40 | 1.06 | 1.83 |
|  | IGHV4-34 | 0.000 | 1.69 | 1.28 | 2.22 |
|  | PLTP | 0.001 | 1.56 | 1.19 | 2.04 |
|  | IGLV2-14 | 0.006 | 1.47 | 1.12 | 1.93 |
|  | CD79A | 0.008 | 1.45 | 1.10 | 1.90 |
|  | ASNS | 0.000 | 1.91 | 1.46 | 2.52 |
|  | IGLV2-11 | 0.011 | 1.43 | 1.09 | 1.87 |
|  | MICB | 0.000 | 1.98 | 1.50 | 2.62 |
|  | MYDGF | 0.000 | 1.96 | 1.48 | 2.60 |
|  | PML | 0.000 | 1.67 | 1.27 | 2.19 |
|  | TAZ | 0.001 | 1.61 | 1.23 | 2.11 |
|  | IL15RA | 0.000 | 1.86 | 1.40 | 2.47 |
|  | NFKBIE | 0.023 | 1.37 | 1.04 | 1.80 |
|  | AURKA | 0.000 | 1.88 | 1.43 | 2.48 |
|  | S100A11 | 0.004 | 1.49 | 1.14 | 1.94 |
|  | IL34 | 0.000 | 1.68 | 1.28 | 2.21 |
|  | CXCL8 | 0.030 | 1.35 | 1.03 | 1.76 |
|  | IFNAR2 | 0.002 | 1.55 | 1.18 | 2.04 |
|  | IGHV3-74 | 0.001 | 1.59 | 1.21 | 2.09 |
|  | LCK | 0.003 | 1.51 | 1.15 | 1.99 |
|  | SDC3 | 0.014 | 1.41 | 1.07 | 1.84 |
|  | BID | 0.000 | 1.95 | 1.47 | 2.58 |
|  | SEM1 | 0.002 | 1.52 | 1.16 | 1.99 |
|  | AKR1C1 | 0.000 | 0.53 | 0.40 | 0.71 |
|  | IGKV1-9 | 0.001 | 1.57 | 1.19 | 2.07 |
|  | IKBKE | 0.004 | 1.48 | 1.13 | 1.94 |
| downregulated | MTOR | 0.013 | 0.70 | 0.54 | 0.93 |
|  | SSTR1 | 0.000 | 0.56 | 0.43 | 0.73 |
|  | PPARA | 0.000 | 0.52 | 0.40 | 0.69 |
|  | PEBP1 | 0.000 | 0.62 | 0.47 | 0.81 |
|  | LTBP1 | 0.010 | 1.44 | 1.09 | 1.89 |
|  | ACO1 | 0.004 | 0.67 | 0.51 | 0.88 |
|  | TKFC | 0.000 | 0.61 | 0.46 | 0.80 |
|  | PTGDS | 0.000 | 1.69 | 1.28 | 2.23 |
|  | VAV3 | 0.001 | 0.64 | 0.49 | 0.84 |
|  | GDF7 | 0.001 | 0.64 | 0.49 | 0.84 |
|  | FGF7 | 0.000 | 1.67 | 1.27 | 2.20 |
|  | SORT1 | 0.007 | 0.69 | 0.52 | 0.90 |
|  | GABARAPL1 | 0.004 | 0.67 | 0.51 | 0.88 |
|  | MUC1 | 0.041 | 1.32 | 1.01 | 1.73 |
|  | PRKCQ | 0.009 | 0.70 | 0.53 | 0.91 |
|  | CAT | 0.001 | 0.63 | 0.48 | 0.83 |
|  | BPHL | 0.000 | 0.44 | 0.33 | 0.58 |
|  | CDH1 | 0.040 | 0.76 | 0.58 | 0.99 |
|  | NOX4 | 0.009 | 0.70 | 0.53 | 0.91 |
|  | PTGS2 | 0.007 | 1.45 | 1.11 | 1.90 |
|  | APOM | 0.009 | 0.70 | 0.53 | 0.91 |
|  | KL | 0.000 | 0.40 | 0.30 | 0.53 |
|  | TGFBR3 | 0.005 | 0.68 | 0.52 | 0.89 |
|  | CMTM4 | 0.001 | 0.64 | 0.48 | 0.84 |
|  | PTGFR | 0.009 | 1.43 | 1.09 | 1.87 |
|  | AZGP1 | 0.001 | 0.63 | 0.48 | 0.82 |
|  | TEK | 0.001 | 0.64 | 0.49 | 0.84 |
|  | LINC00472 | 0.011 | 0.70 | 0.54 | 0.92 |
|  | THRB | 0.000 | 0.61 | 0.47 | 0.81 |
|  | NR3C2 | 0.000 | 0.51 | 0.39 | 0.67 |
|  | MIOX | 0.000 | 0.55 | 0.42 | 0.73 |
|  | DES | 0.014 | 1.40 | 1.07 | 1.84 |
|  | SEMA3G | 0.015 | 0.72 | 0.55 | 0.94 |
|  | PSAT1 | 0.000 | 2.14 | 1.62 | 2.83 |
|  | PTH1R | 0.000 | 0.48 | 0.36 | 0.65 |
|  | BMPR1B | 0.000 | 1.65 | 1.25 | 2.17 |
|  | PLXNA4 | 0.031 | 1.34 | 1.03 | 1.76 |
|  | GREM1 | 0.000 | 2.04 | 1.54 | 2.70 |
|  | PCK2 | 0.001 | 0.62 | 0.47 | 0.81 |
|  | PDGFRA | 0.004 | 1.48 | 1.13 | 1.94 |
|  | SEMA6D | 0.008 | 0.69 | 0.53 | 0.91 |
|  | ESRRG | 0.000 | 0.61 | 0.47 | 0.80 |
|  | PROM2 | 0.000 | 2.07 | 1.56 | 2.74 |
|  | FAM3B | 0.033 | 1.34 | 1.02 | 1.75 |
|  | MT1G | 0.000 | 1.70 | 1.29 | 2.23 |

# Supplementary Table 3 Difference immune cells and factors between KC patients with low-risk value and high-risk value in training group

| cell_type | Low (n=57) | | High (n=34) | |
| --- | --- | --- | --- | --- |
|  | Mean | SD | Mean | SD |
| uncharacterized cell_QUANTISEQ | 0.63 | 0.06 | 0.73 | 0.10 |
| B cell_QUANTISEQ | 0.02 | 0.01 | 0.01 | 0.00 |
| Neutrophil_QUANTISEQ | 0.20 | 0.06 | 0.14 | 0.08 |
| NK cell_QUANTISEQ | 0.02 | 0.00 | 0.02 | 0.01 |
| T cell CD4+ (non-regulatory)_QUANTISEQ | 0.01 | 0.02 | 0.00 | 0.01 |
| Macrophage M2_QUANTISEQ | 0.06 | 0.02 | 0.04 | 0.02 |
| Myeloid dendritic cell_QUANTISEQ | 0.01 | 0.01 | 0.01 | 0.01 |
| uncharacterized cell_EPIC | 0.39 | 0.24 | 0.59 | 0.18 |
| Endothelial cell_EPIC | 0.44 | 0.24 | 0.26 | 0.17 |
| T cell CD4+_EPIC | 0.07 | 0.03 | 0.05 | 0.03 |
| NK cell_EPIC | 0.00 | 0.00 | 0.00 | 0.00 |
| Endothelial cell_MCPCOUNTER | 3101.61 | 1349.79 | 2286.52 | 1218.37 |
| Myeloid dendritic cell_MCPCOUNTER | 182.04 | 79.49 | 140.77 | 63.78 |
| T cell regulatory (Tregs)_CIBERSORT | 0.00 | 0.01 | 0.01 | 0.02 |
| Myeloid dendritic cell resting_CIBERSORT | 0.00 | 0.01 | 0.00 | 0.00 |
| Macrophage M1_CIBERSORT | 0.06 | 0.04 | 0.04 | 0.04 |
| T cell regulatory (Tregs)_CIBERSORT-ABS | 0.03 | 0.08 | 0.14 | 0.23 |
| NK cell activated_CIBERSORT-ABS | 0.25 | 0.24 | 0.43 | 0.41 |
| Myeloid dendritic cell resting_CIBERSORT-ABS | 0.05 | 0.11 | 0.00 | 0.01 |
| T cell CD8+_TIMER | 0.26 | 0.11 | 0.16 | 0.12 |
| T cell NK_XCELL | 0.04 | 0.04 | 0.12 | 0.09 |
| T cell CD4+ Th1_XCELL | 0.03 | 0.03 | 0.07 | 0.04 |
| Hematopoietic stem cell_XCELL | 0.52 | 0.16 | 0.38 | 0.19 |
| B cell plasma_XCELL | 0.00 | 0.00 | 0.01 | 0.02 |
| T cell CD4+ central memory_XCELL | 0.02 | 0.02 | 0.05 | 0.05 |
| Granulocyte-monocyte progenitor_XCELL | 0.02 | 0.02 | 0.01 | 0.01 |
| T cell regulatory (Tregs)_XCELL | 0.00 | 0.01 | 0.00 | 0.00 |

# Supplementary Table 4 Difference immune cells and factors between KC patients with low-risk value and high-risk value in validation group.

| cell_type | Low (n=742) | | High (n=76) | |
| --- | --- | --- | --- | --- |
|  | Mean | SD | Mean | SD |
| T cell regulatory (Tregs)_CIBERSORT | 0.01 | 0.02 | 0.03 | 0.03 |
| T cell follicular helper_CIBERSORT | 0.02 | 0.02 | 0.04 | 0.03 |
| Macrophage M2_CIBERSORT | 0.36 | 0.13 | 0.29 | 0.14 |
| Macrophage M0_CIBERSORT | 0.02 | 0.05 | 0.05 | 0.11 |
| T cell CD4+ memory resting_CIBERSORT | 0.15 | 0.08 | 0.12 | 0.08 |
| NK cell activated_CIBERSORT | 0.05 | 0.03 | 0.07 | 0.04 |
| B cell naive_CIBERSORT | 0.01 | 0.02 | 0.02 | 0.04 |
| Neutrophil_MCPCOUNTER | 12.81 | 5.45 | 8.98 | 4.85 |
| Endothelial cell_MCPCOUNTER | 37.38 | 33.61 | 16.41 | 14.53 |
| Monocyte_MCPCOUNTER | 35.81 | 16.75 | 25.24 | 21.90 |
| Macrophage/Monocyte_MCPCOUNTER | 35.81 | 16.75 | 25.24 | 21.90 |
| Endothelial cell_EPIC | 0.14 | 0.13 | 0.06 | 0.05 |
| Cancer associated fibroblast_EPIC | 0.02 | 0.03 | 0.04 | 0.10 |
| uncharacterized cell_EPIC | 0.74 | 0.15 | 0.81 | 0.12 |
| T cell CD8+_EPIC | 0.03 | 0.03 | 0.02 | 0.02 |
| T cell CD4+_EPIC | 0.04 | 0.02 | 0.04 | 0.03 |
| NK cell_EPIC | 0.00 | 0.00 | 0.00 | 0.00 |
| Macrophage_TIMER | 0.08 | 0.11 | 0.05 | 0.12 |
| B cell_TIMER | 0.10 | 0.08 | 0.08 | 0.09 |
| Neutrophil_QUANTISEQ | 0.11 | 0.05 | 0.06 | 0.05 |
| B cell_QUANTISEQ | 0.01 | 0.01 | 0.01 | 0.02 |
| T cell CD4+ (non-regulatory)_QUANTISEQ | 0.04 | 0.02 | 0.02 | 0.02 |
| uncharacterized cell_QUANTISEQ | 0.70 | 0.09 | 0.76 | 0.11 |
| Myeloid dendritic cell_QUANTISEQ | 0.01 | 0.01 | 0.00 | 0.01 |
| Monocyte_QUANTISEQ | 0.00 | 0.01 | 0.01 | 0.03 |
| Macrophage M1_QUANTISEQ | 0.03 | 0.02 | 0.04 | 0.04 |
| NK cell_QUANTISEQ | 0.01 | 0.01 | 0.01 | 0.01 |
| Macrophage M2_QUANTISEQ | 0.05 | 0.03 | 0.04 | 0.04 |
| T cell regulatory (Tregs)_QUANTISEQ | 0.01 | 0.01 | 0.01 | 0.01 |
| Macrophage M2_CIBERSORT-ABS | 0.17 | 0.10 | 0.12 | 0.10 |
| T cell regulatory (Tregs)_CIBERSORT-ABS | 0.01 | 0.01 | 0.01 | 0.01 |
| T cell follicular helper_CIBERSORT-ABS | 0.01 | 0.02 | 0.02 | 0.02 |
| Macrophage M0_CIBERSORT-ABS | 0.01 | 0.02 | 0.02 | 0.03 |
| T cell CD4+ Th2_XCELL | 0.04 | 0.05 | 0.10 | 0.13 |
| T cell NK_XCELL | 0.07 | 0.05 | 0.12 | 0.09 |
| B cell memory_XCELL | 0.00 | 0.01 | 0.01 | 0.04 |
| B cell_XCELL | 0.02 | 0.03 | 0.05 | 0.10 |
| B cell naive_XCELL | 0.00 | 0.00 | 0.00 | 0.01 |
| T cell CD4+ effector memory_XCELL | 0.06 | 0.04 | 0.04 | 0.04 |
| T cell CD4+ Th1_XCELL | 0.11 | 0.09 | 0.16 | 0.13 |
| Endothelial cell_XCELL | 0.17 | 0.14 | 0.09 | 0.08 |
| B cell plasma_XCELL | 0.01 | 0.01 | 0.02 | 0.03 |
| Class-switched memory B cell_XCELL | 0.01 | 0.01 | 0.01 | 0.03 |
| Macrophage M2_XCELL | 0.05 | 0.04 | 0.03 | 0.03 |
| Hematopoietic stem cell_XCELL | 0.18 | 0.16 | 0.11 | 0.11 |
| T cell CD4+ naive_XCELL | 0.00 | 0.01 | 0.01 | 0.02 |
| Plasmacytoid dendritic cell_XCELL | 0.01 | 0.01 | 0.01 | 0.02 |
| Common lymphoid progenitor_XCELL | 0.02 | 0.02 | 0.03 | 0.02 |
| immune score_XCELL | 0.11 | 0.10 | 0.14 | 0.16 |

# Supplementary Table 5 Difference immune cells and factors between KC patients with low-risk value and high-risk value in entire group.

| cell_type | Low (n=799) | | High (n=110) | |
| --- | --- | --- | --- | --- |
|  | Mean | SD | Mean | SD |
| T cell CD4+ (non-regulatory)_QUANTISEQ | 0.03 | 0.02 | 0.01 | 0.02 |
| Neutrophil_QUANTISEQ | 0.12 | 0.05 | 0.09 | 0.07 |
| Macrophage M2_QUANTISEQ | 0.05 | 0.03 | 0.04 | 0.03 |
| Monocyte_QUANTISEQ | 0.00 | 0.01 | 0.01 | 0.02 |
| Cancer associated fibroblast_EPIC | 0.02 | 0.03 | 0.04 | 0.09 |
| Endothelial cell_EPIC | 0.16 | 0.16 | 0.12 | 0.14 |
| T cell regulatory (Tregs)_CIBERSORT-ABS | 0.01 | 0.02 | 0.05 | 0.14 |
| Monocyte_CIBERSORT-ABS | 0.06 | 0.18 | 0.21 | 0.39 |
| T cell CD4+ memory resting_CIBERSORT-ABS | 0.22 | 0.62 | 0.68 | 1.06 |
| T cell CD8+_CIBERSORT-ABS | 0.17 | 0.48 | 0.54 | 1.11 |
| B cell naive_CIBERSORT-ABS | 0.02 | 0.08 | 0.09 | 0.24 |
| Mast cell activated_CIBERSORT-ABS | 0.05 | 0.15 | 0.14 | 0.32 |
| B cell memory_CIBERSORT-ABS | 0.00 | 0.01 | 0.01 | 0.03 |
| B cell plasma_CIBERSORT-ABS | 0.03 | 0.05 | 0.09 | 0.32 |
| T cell follicular helper_CIBERSORT-ABS | 0.02 | 0.04 | 0.04 | 0.11 |
| T cell CD4+ naive_CIBERSORT-ABS | 0.00 | 0.00 | 0.00 | 0.00 |
| Eosinophil_CIBERSORT-ABS | 0.00 | 0.00 | 0.00 | 0.01 |
| T cell regulatory (Tregs)_CIBERSORT | 0.01 | 0.02 | 0.02 | 0.03 |
| Macrophage M2_CIBERSORT | 0.36 | 0.13 | 0.30 | 0.13 |
| B cell naive_CIBERSORT | 0.01 | 0.02 | 0.02 | 0.04 |
| Macrophage M0_CIBERSORT | 0.02 | 0.05 | 0.04 | 0.10 |
| T cell follicular helper_CIBERSORT | 0.02 | 0.02 | 0.03 | 0.03 |
| Eosinophil_CIBERSORT | 0.00 | 0.00 | 0.00 | 0.00 |
| NK cell activated_CIBERSORT | 0.05 | 0.03 | 0.06 | 0.04 |
| Macrophage/Monocyte_MCPCOUNTER | 199.05 | 624.09 | 848.56 | 1392.16 |
| Monocyte_MCPCOUNTER | 199.05 | 624.09 | 848.56 | 1392.16 |
| T cell_MCPCOUNTER | 31.65 | 61.24 | 87.40 | 125.16 |
| Cancer associated fibroblast_MCPCOUNTER | 530.88 | 1782.90 | 2445.66 | 5281.96 |
| cytotoxicity score_MCPCOUNTER | 24.22 | 76.21 | 88.95 | 170.79 |
| Neutrophil_MCPCOUNTER | 60.76 | 182.14 | 192.52 | 300.45 |
| Myeloid dendritic cell_MCPCOUNTER | 16.36 | 50.61 | 45.63 | 72.97 |
| B cell_MCPCOUNTER | 8.85 | 31.93 | 82.64 | 409.63 |
| Macrophage_TIMER | 0.08 | 0.11 | 0.05 | 0.11 |
| T cell NK_XCELL | 0.07 | 0.05 | 0.12 | 0.09 |
| T cell CD4+ central memory_XCELL | 0.01 | 0.01 | 0.02 | 0.04 |
| T cell CD4+ Th2_XCELL | 0.03 | 0.05 | 0.07 | 0.12 |
| Macrophage M2_XCELL | 0.04 | 0.04 | 0.02 | 0.03 |
| B cell memory_XCELL | 0.00 | 0.01 | 0.01 | 0.03 |
| Endothelial cell_XCELL | 0.17 | 0.14 | 0.10 | 0.08 |
| B cell_XCELL | 0.02 | 0.03 | 0.04 | 0.09 |
| B cell naive_XCELL | 0.00 | 0.00 | 0.00 | 0.01 |
| B cell plasma_XCELL | 0.01 | 0.01 | 0.01 | 0.03 |
| T cell CD4+ naive_XCELL | 0.00 | 0.01 | 0.01 | 0.02 |
| T cell CD4+ effector memory_XCELL | 0.06 | 0.04 | 0.05 | 0.04 |
| stroma score_XCELL | 0.10 | 0.07 | 0.07 | 0.06 |
| T cell CD4+ Th1_XCELL | 0.10 | 0.09 | 0.13 | 0.12 |
| Class-switched memory B cell_XCELL | 0.01 | 0.01 | 0.01 | 0.02 |
| T cell CD4+ memory_XCELL | 0.00 | 0.01 | 0.01 | 0.01 |
| Plasmacytoid dendritic cell_XCELL | 0.01 | 0.01 | 0.01 | 0.02 |

# Supplementary Table 6 Significantly enriched GO term in training group.

| Description | enrichmentScore | pvalue |
| --- | --- | --- |
| BP_phosphorylation | 0.36 | 0.001 |
| BP_response to abiotic stimulus | 0.42 | 0.001 |
| BP_generation of precursor metabolites and energy | 0.56 | 0.001 |
| BP_nucleoside phosphate metabolic process | 0.43 | 0.001 |
| BP_purine nucleotide metabolic process | 0.51 | 0.001 |
| BP_purine ribonucleotide metabolic process | 0.51 | 0.001 |
| BP_ribonucleotide metabolic process | 0.51 | 0.001 |
| BP_cation transport | 0.43 | 0.001 |
| BP_drug metabolic process | 0.49 | 0.001 |
| BP_nucleobase-containing small molecule metabolic process | 0.41 | 0.001 |
| BP_nucleoside triphosphate metabolic process | 0.56 | 0.001 |
| BP_purine nucleoside triphosphate metabolic process | 0.56 | 0.001 |
| BP_ribonucleoside triphosphate metabolic process | 0.56 | 0.001 |
| BP_purine ribonucleoside triphosphate metabolic process | 0.56 | 0.001 |
| BP_ribose phosphate metabolic process | 0.50 | 0.001 |
| BP_purine-containing compound metabolic process | 0.47 | 0.001 |
| BP_nucleoside monophosphate metabolic process | 0.61 | 0.001 |
| BP_purine nucleoside monophosphate metabolic process | 0.62 | 0.001 |
| BP_ribonucleoside monophosphate metabolic process | 0.62 | 0.001 |
| BP_purine ribonucleoside monophosphate metabolic process | 0.62 | 0.001 |
| BP_energy derivation by oxidation of organic compounds | 0.55 | 0.001 |
| BP_ATP metabolic process | 0.64 | 0.001 |
| BP_oxidative phosphorylation | 0.71 | 0.001 |
| BP_acute inflammatory response | 0.62 | 0.001 |
| BP_electron transport chain | 0.76 | 0.001 |
| BP_cellular respiration | 0.75 | 0.001 |
| BP_respiratory electron transport chain | 0.79 | 0.001 |
| BP_ATP synthesis coupled electron transport | 0.79 | 0.001 |
| BP_mitochondrial ATP synthesis coupled electron transport | 0.79 | 0.001 |
| BP_NADH dehydrogenase complex assembly | 0.66 | 0.001 |
| BP_mitochondrial respiratory chain complex I assembly | 0.66 | 0.001 |
| BP_mitochondrial respiratory chain complex assembly | 0.66 | 0.001 |
| BP_organophosphate metabolic process | 0.38 | 0.002 |
| BP_nucleotide metabolic process | 0.44 | 0.002 |
| BP_monovalent inorganic cation transport | 0.56 | 0.003 |
| BP_heart development | -0.48 | 0.004 |
| BP_response to growth factor | -0.50 | 0.005 |
| BP_cellular response to growth factor stimulus | -0.50 | 0.005 |
| BP_cation transmembrane transport | 0.49 | 0.005 |
| BP_blood vessel morphogenesis | -0.36 | 0.006 |
| BP_blood vessel development | -0.34 | 0.006 |
| BP_enzyme linked receptor protein signaling pathway | -0.28 | 0.006 |
| BP_vasculature development | -0.36 | 0.006 |
| BP_cardiovascular system development | -0.36 | 0.006 |
| BP_tube morphogenesis | -0.31 | 0.007 |
| BP_circulatory system development | -0.31 | 0.007 |
| BP_response to carbohydrate | -0.54 | 0.007 |
| BP_smooth muscle cell proliferation | -0.49 | 0.007 |
| BP_regulation of smooth muscle cell proliferation | -0.49 | 0.007 |
| BP_response to antibiotic | 0.51 | 0.007 |
| BP_regulation of response to external stimulus | -0.26 | 0.007 |
| BP_positive regulation of vasculature development | -0.41 | 0.008 |
| BP_response to stress | 0.29 | 0.008 |
| BP_regulation of vasculature development | -0.42 | 0.009 |
| BP_response to oxidative stress | 0.48 | 0.009 |
| BP_system process | -0.22 | 0.009 |
| BP_response to inorganic substance | 0.40 | 0.010 |
| BP_regulation of metal ion transport | 0.56 | 0.010 |
| BP_proton transmembrane transport | 0.54 | 0.010 |
| BP_mitochondrion organization | 0.46 | 0.010 |
| BP_regulation of epithelial cell migration | -0.46 | 0.011 |
| BP_steroid metabolic process | -0.42 | 0.011 |
| BP_protein kinase B signaling | -0.41 | 0.011 |
| BP_positive regulation of multicellular organismal process | -0.23 | 0.012 |
| BP_ion transport | 0.34 | 0.012 |
| BP_phosphate-containing compound metabolic process | 0.29 | 0.012 |
| BP_regulation of angiogenesis | -0.39 | 0.013 |
| BP_ion transmembrane transport | 0.38 | 0.013 |
| BP_tube development | -0.27 | 0.013 |
| BP_receptor-mediated endocytosis | 0.51 | 0.014 |
| BP_cellular response to endogenous stimulus | -0.24 | 0.014 |
| BP_negative regulation of molecular function | 0.39 | 0.014 |
| BP_muscle organ development | -0.43 | 0.014 |
| BP_striated muscle tissue development | -0.43 | 0.014 |
| BP_muscle tissue development | -0.43 | 0.014 |
| BP_phosphorus metabolic process | 0.28 | 0.014 |
| BP_positive regulation of response to stimulus | -0.20 | 0.014 |
| BP_response to oxygen levels | 0.45 | 0.015 |
| BP_angiogenesis | -0.34 | 0.016 |
| BP_muscle cell proliferation | -0.44 | 0.018 |
| BP_peptide biosynthetic process | -0.40 | 0.018 |
| BP_cell-cell signaling | -0.24 | 0.020 |
| BP_regulation of inflammatory response | -0.27 | 0.020 |
| BP_positive regulation of transcription by RNA polymerase II | -0.33 | 0.021 |
| BP_regulation of multicellular organismal development | -0.19 | 0.021 |
| BP_epithelial cell migration | -0.40 | 0.022 |
| BP_tissue migration | -0.40 | 0.022 |
| BP_epithelium migration | -0.40 | 0.022 |
| BP_regulation of gene expression | -0.18 | 0.022 |
| BP_response to hypoxia | 0.44 | 0.022 |
| BP_regulation of multicellular organismal process | -0.21 | 0.023 |
| BP_inorganic cation transmembrane transport | 0.46 | 0.023 |
| BP_response to drug | 0.36 | 0.024 |
| BP_regulation of biological quality | -0.17 | 0.024 |
| BP_positive regulation of epithelial cell migration | -0.43 | 0.024 |
| BP_response to oxygen-containing compound | 0.31 | 0.024 |
| BP_circulatory system process | -0.35 | 0.026 |
| BP_blood circulation | -0.35 | 0.026 |
| BP_tissue development | -0.21 | 0.027 |
| BP_regulation of anatomical structure morphogenesis | -0.27 | 0.027 |
| BP_positive regulation of cell death | 0.44 | 0.027 |
| BP_regulation of protein kinase B signaling | -0.42 | 0.028 |
| BP_positive regulation of angiogenesis | -0.38 | 0.029 |
| BP_response to chemical | 0.25 | 0.030 |
| BP_amide biosynthetic process | -0.35 | 0.030 |
| BP_regulation of MAP kinase activity | 0.48 | 0.032 |
| BP_translation | -0.39 | 0.032 |
| BP_organic anion transport | -0.34 | 0.032 |
| BP_inorganic ion transmembrane transport | 0.43 | 0.032 |
| BP_response to toxic substance | 0.41 | 0.033 |
| BP_anion transport | -0.31 | 0.033 |
| BP_negative regulation of intracellular signal transduction | 0.51 | 0.034 |
| BP_response to mechanical stimulus | -0.40 | 0.036 |
| BP_response to decreased oxygen levels | 0.41 | 0.036 |
| BP_negative regulation of catalytic activity | 0.37 | 0.036 |
| BP_negative regulation of cell communication | 0.35 | 0.036 |
| BP_negative regulation of signaling | 0.35 | 0.036 |
| BP_regulation of response to stress | -0.19 | 0.038 |
| BP_cellular response to organic substance | -0.14 | 0.038 |
| BP_organic acid transport | -0.33 | 0.039 |
| BP_carboxylic acid transport | -0.33 | 0.039 |
| BP_establishment of protein localization to organelle | -0.38 | 0.039 |
| BP_chemical homeostasis | 0.34 | 0.040 |
| BP_cellular ion homeostasis | 0.42 | 0.040 |
| BP_cellular metal ion homeostasis | 0.42 | 0.040 |
| BP_cellular cation homeostasis | 0.42 | 0.040 |
| BP_transmembrane transport | 0.32 | 0.041 |
| BP_positive regulation of peptide secretion | 0.49 | 0.044 |
| BP_oxidation-reduction process | 0.33 | 0.044 |
| BP_positive regulation of developmental process | -0.19 | 0.044 |
| BP_carbohydrate derivative metabolic process | 0.30 | 0.046 |
| BP_cellular calcium ion homeostasis | 0.46 | 0.047 |
| CC_mitochondrion | 0.40 | 0.001 |
| CC_organelle envelope | 0.44 | 0.001 |
| CC_envelope | 0.44 | 0.001 |
| CC_mitochondrial part | 0.48 | 0.001 |
| CC_mitochondrial envelope | 0.52 | 0.001 |
| CC_mitochondrial membrane | 0.52 | 0.001 |
| CC_membrane protein complex | 0.55 | 0.001 |
| CC_mitochondrial inner membrane | 0.60 | 0.001 |
| CC_organelle inner membrane | 0.60 | 0.001 |
| CC_mitochondrial membrane part | 0.67 | 0.001 |
| CC_mitochondrial protein complex | 0.66 | 0.001 |
| CC_inner mitochondrial membrane protein complex | 0.69 | 0.001 |
| CC_respiratory chain | 0.83 | 0.001 |
| CC_oxidoreductase complex | 0.75 | 0.001 |
| CC_respiratory chain complex | 0.82 | 0.001 |
| CC_mitochondrial respiratory chain | 0.80 | 0.001 |
| CC_organelle membrane | 0.32 | 0.003 |
| CC_cell body | 0.50 | 0.004 |
| CC_vesicle lumen | 0.45 | 0.008 |
| CC_cytoplasmic vesicle lumen | 0.45 | 0.008 |
| CC_membrane part | 0.27 | 0.009 |
| CC_cell surface | -0.36 | 0.012 |
| CC_integral component of plasma membrane | -0.32 | 0.012 |
| CC_membrane | 0.25 | 0.013 |
| CC_plasma membrane part | -0.30 | 0.013 |
| CC_intrinsic component of plasma membrane | -0.32 | 0.014 |
| CC_integral component of membrane | 0.28 | 0.014 |
| CC_neuronal cell body | 0.49 | 0.016 |
| CC_intrinsic component of membrane | 0.27 | 0.019 |
| CC_membrane raft | -0.50 | 0.024 |
| CC_membrane region | -0.50 | 0.024 |
| CC_membrane microdomain | -0.50 | 0.024 |
| CC_protein-containing complex | 0.28 | 0.025 |
| CC_cell junction | -0.25 | 0.026 |
| CC_plasma membrane | -0.23 | 0.028 |
| CC_cell periphery | -0.23 | 0.029 |
| CC_microtubule organizing center | -0.38 | 0.031 |
| CC_external side of plasma membrane | -0.38 | 0.035 |
| CC_catalytic complex | 0.36 | 0.041 |
| MF_oxidoreductase activity | 0.47 | 0.001 |
| MF_oxidoreductase activity, acting on NAD(P)H | 0.72 | 0.001 |
| MF_oxidoreductase activity, acting on NAD(P)H, quinone or similar compound as acceptor | 0.72 | 0.001 |
| MF_enzyme inhibitor activity | 0.58 | 0.004 |
| MF_inorganic cation transmembrane transporter activity | 0.57 | 0.005 |
| MF_signaling receptor activity | -0.34 | 0.006 |
| MF_molecular transducer activity | -0.34 | 0.006 |
| MF_ion transmembrane transporter activity | 0.47 | 0.007 |
| MF_inorganic molecular entity transmembrane transporter activity | 0.47 | 0.007 |
| MF_transmembrane transporter activity | 0.42 | 0.007 |
| MF_cation transmembrane transporter activity | 0.54 | 0.008 |
| MF_transmembrane signaling receptor activity | -0.30 | 0.011 |
| MF_catalytic activity, acting on a protein | -0.24 | 0.014 |
| MF_transporter activity | 0.39 | 0.018 |
| MF_small molecule binding | -0.19 | 0.027 |
| MF_calcium ion binding | -0.29 | 0.030 |
| MF_hydrolase activity, acting on ester bonds | -0.40 | 0.039 |

**Supplementary Table 7 Enriched KEGG term in training group.**

| Description | enrichmentScore | pvalue |
| --- | --- | --- |
| Diabetic cardiomyopathy | 0.67 | 0.001 |
| Thermogenesis | 0.69 | 0.001 |
| Alzheimer disease | 0.75 | 0.001 |
| Pathways of neurodegeneration - multiple diseases | 0.79 | 0.001 |
| Oxidative phosphorylation | 0.78 | 0.001 |
| Parkinson disease | 0.79 | 0.001 |
| Amyotrophic lateral sclerosis | 0.78 | 0.001 |
| Huntington disease | 0.77 | 0.001 |
| Prion disease | 0.79 | 0.001 |
| Chemical carcinogenesis - reactive oxygen species | 0.78 | 0.001 |
| Retrograde endocannabinoid signaling | 0.73 | 0.001 |
| Metabolic pathways | 0.32 | 0.013 |
| Proteoglycans in cancer | -0.44 | 0.032 |
| PI3K-Akt signaling pathway | -0.31 | 0.041 |
| Coronavirus disease - COVID-19 | -0.43 | 0.043 |
| Pathways in cancer | -0.31 | 0.045 |
| Neuroactive ligand-receptor interaction | -0.29 | 0.139 |
| MAPK signaling pathway | -0.32 | 0.143 |
| Human papillomavirus infection | -0.36 | 0.146 |

**Supplementary Table 8 Significantly enriched GO term in validation group.**

| Description | enrichmentScore | pvalue |
| --- | --- | --- |
| BP_cellular component organization or biogenesis | 0.22 | 0.001 |
| BP_cellular component organization | 0.23 | 0.001 |
| BP_negative regulation of biological process | 0.22 | 0.001 |
| BP_regulation of cellular metabolic process | 0.24 | 0.001 |
| BP_positive regulation of cellular process | 0.21 | 0.001 |
| BP_cell surface receptor signaling pathway | 0.27 | 0.001 |
| BP_positive regulation of macromolecule metabolic process | 0.23 | 0.001 |
| BP_amide transport | 0.29 | 0.001 |
| BP_establishment of protein localization | 0.31 | 0.001 |
| BP_positive regulation of protein metabolic process | 0.28 | 0.001 |
| BP_cell cycle | 0.32 | 0.001 |
| BP_cell death | 0.28 | 0.001 |
| BP_peptide transport | 0.30 | 0.001 |
| BP_programmed cell death | 0.27 | 0.001 |
| BP_protein localization | 0.29 | 0.001 |
| BP_protein transport | 0.31 | 0.002 |
| BP_regulation of cell cycle | 0.34 | 0.002 |
| BP_cell division | 0.46 | 0.002 |
| BP_inner ear development | 0.51 | 0.002 |
| BP_chloride transport | 0.72 | 0.002 |
| BP_monovalent inorganic cation homeostasis | 0.62 | 0.002 |
| BP_regulation of pH | 0.74 | 0.002 |
| BP_transition metal ion transport | 0.74 | 0.002 |
| BP_chloride transmembrane transport | 0.75 | 0.002 |
| BP_cellular monovalent inorganic cation homeostasis | 0.72 | 0.002 |
| BP_iron ion transport | 0.81 | 0.002 |
| BP_regulation of cellular pH | 0.75 | 0.002 |
| BP_regulation of intracellular pH | 0.75 | 0.002 |
| BP_carbohydrate catabolic process | -0.70 | 0.002 |
| BP_protein targeting | -0.58 | 0.002 |
| BP_monocarboxylic acid catabolic process | -0.58 | 0.002 |
| BP_anatomical structure development | 0.20 | 0.003 |
| BP_positive regulation of biological process | 0.20 | 0.003 |
| BP_vesicle-mediated transport | 0.27 | 0.003 |
| BP_macromolecule localization | 0.24 | 0.003 |
| BP_positive regulation of signal transduction | 0.27 | 0.003 |
| BP_positive regulation of signaling | 0.26 | 0.003 |
| BP_positive regulation of cellular metabolic process | 0.23 | 0.003 |
| BP_cell development | 0.24 | 0.003 |
| BP_positive regulation of nitrogen compound metabolic process | 0.23 | 0.003 |
| BP_regulation of cellular component organization | 0.23 | 0.003 |
| BP_regulation of protein metabolic process | 0.24 | 0.003 |
| BP_positive regulation of response to stimulus | 0.24 | 0.003 |
| BP_regulation of cellular protein metabolic process | 0.24 | 0.003 |
| BP_transmembrane transport | -0.27 | 0.003 |
| BP_positive regulation of cellular component organization | 0.28 | 0.003 |
| BP_enzyme linked receptor protein signaling pathway | 0.30 | 0.003 |
| BP_transmembrane receptor protein tyrosine kinase signaling pathway | 0.38 | 0.003 |
| BP_growth | 0.31 | 0.003 |
| BP_inorganic ion transmembrane transport | 0.36 | 0.003 |
| BP_organic acid metabolic process | -0.32 | 0.003 |
| BP_small molecule metabolic process | -0.26 | 0.003 |
| BP_cellular ion homeostasis | 0.37 | 0.003 |
| BP_ear development | 0.49 | 0.003 |
| BP_positive regulation of growth | 0.43 | 0.003 |
| BP_inner ear morphogenesis | 0.59 | 0.003 |
| BP_ear morphogenesis | 0.57 | 0.003 |
| BP_cellular response to stimulus | 0.19 | 0.004 |
| BP_regulation of macromolecule metabolic process | 0.22 | 0.004 |
| BP_negative regulation of cellular process | 0.21 | 0.004 |
| BP_apoptotic process | 0.25 | 0.004 |
| BP_positive regulation of cellular protein metabolic process | 0.27 | 0.004 |
| BP_regulation of developmental process | 0.23 | 0.004 |
| BP_positive regulation of cell communication | 0.26 | 0.004 |
| BP_regulation of catalytic activity | 0.25 | 0.004 |
| BP_mitotic cell cycle process | 0.34 | 0.005 |
| BP_regulation of kinase activity | 0.33 | 0.005 |
| BP_inorganic ion homeostasis | 0.34 | 0.005 |
| BP_chromosome organization | 0.35 | 0.005 |
| BP_positive regulation of cell death | 0.37 | 0.005 |
| BP_regulation of growth | 0.34 | 0.005 |
| BP_cation homeostasis | 0.35 | 0.005 |
| BP_xenobiotic metabolic process | -0.54 | 0.005 |
| BP_drug transmembrane transport | -0.51 | 0.005 |
| BP_sensory organ development | 0.37 | 0.005 |
| BP_alpha-amino acid catabolic process | -0.53 | 0.005 |
| BP_lipid modification | -0.53 | 0.005 |
| BP_organic acid transmembrane transport | -0.50 | 0.005 |
| BP_carboxylic acid transmembrane transport | -0.50 | 0.005 |
| BP_neurotransmitter transport | -0.45 | 0.005 |
| BP_drug transport | -0.50 | 0.005 |
| BP_inorganic anion transmembrane transport | 0.56 | 0.005 |
| BP_cofactor metabolic process | -0.35 | 0.005 |
| BP_purine-containing compound metabolic process | -0.37 | 0.005 |
| BP_carbohydrate metabolic process | -0.37 | 0.005 |
| BP_small molecule catabolic process | -0.41 | 0.005 |
| BP_signal transduction | 0.20 | 0.005 |
| BP_organic acid transport | -0.41 | 0.005 |
| BP_cellular amide metabolic process | -0.37 | 0.005 |
| BP_carboxylic acid transport | -0.41 | 0.005 |
| BP_protein metabolic process | 0.21 | 0.005 |
| BP_organic anion transport | -0.34 | 0.006 |
| BP_cellular localization | 0.24 | 0.006 |
| BP_anion transport | -0.31 | 0.006 |
| BP_cell cycle process | 0.28 | 0.006 |
| BP_mitotic cell cycle | 0.31 | 0.006 |
| BP_chemotaxis | 0.31 | 0.006 |
| BP_taxis | 0.31 | 0.006 |
| BP_regulation of transferase activity | 0.33 | 0.006 |
| BP_regulation of protein kinase activity | 0.34 | 0.006 |
| BP_positive regulation of apoptotic process | 0.39 | 0.006 |
| BP_negative regulation of cell development | 0.42 | 0.007 |
| BP_signaling | 0.19 | 0.007 |
| BP_appendage development | 0.52 | 0.007 |
| BP_limb development | 0.52 | 0.007 |
| BP_cellular amino acid catabolic process | -0.50 | 0.007 |
| BP_positive regulation of cell growth | 0.52 | 0.007 |
| BP_regulation of blood pressure | -0.46 | 0.007 |
| BP_cellular carbohydrate metabolic process | -0.51 | 0.007 |
| BP_cellular protein modification process | 0.22 | 0.007 |
| BP_protein modification process | 0.22 | 0.007 |
| BP_macromolecule modification | 0.21 | 0.007 |
| BP_defense response | 0.26 | 0.007 |
| BP_neurotransmitter metabolic process | -0.44 | 0.007 |
| BP_organic acid catabolic process | -0.43 | 0.008 |
| BP_carboxylic acid catabolic process | -0.43 | 0.008 |
| BP_cellular homeostasis | 0.30 | 0.008 |
| BP_sodium ion transport | -0.41 | 0.008 |
| BP_ion homeostasis | 0.30 | 0.008 |
| BP_negative regulation of catalytic activity | 0.33 | 0.008 |
| BP_cellular chemical homeostasis | 0.32 | 0.008 |
| BP_cellular amino acid metabolic process | -0.33 | 0.008 |
| BP_cellular macromolecule metabolic process | 0.19 | 0.008 |
| BP_immune response-regulating signaling pathway | 0.42 | 0.008 |
| BP_cellular response to DNA damage stimulus | 0.42 | 0.008 |
| BP_regulation of metabolic process | 0.20 | 0.008 |
| BP_regulation of nitrogen compound metabolic process | 0.21 | 0.008 |
| BP_monocarboxylic acid metabolic process | -0.33 | 0.008 |
| BP_response to stress | 0.21 | 0.008 |
| BP_cellular protein metabolic process | 0.22 | 0.008 |
| BP_drug metabolic process | -0.29 | 0.009 |
| BP_proton transmembrane transport | 0.64 | 0.009 |
| BP_insulin receptor signaling pathway | 0.54 | 0.009 |
| BP_carboxylic acid metabolic process | -0.26 | 0.009 |
| BP_regulation of protein localization | 0.28 | 0.009 |
| BP_organic substance transport | -0.20 | 0.010 |
| BP_cell differentiation | 0.20 | 0.010 |
| BP_mitotic nuclear division | 0.40 | 0.010 |
| BP_chromosome segregation | 0.39 | 0.010 |
| BP_negative regulation of cellular metabolic process | 0.24 | 0.010 |
| BP_spindle organization | 0.53 | 0.011 |
| BP_developmental process | 0.18 | 0.011 |
| BP_organic hydroxy compound metabolic process | -0.31 | 0.011 |
| BP_positive regulation of transferase activity | 0.32 | 0.011 |
| BP_regulation of vesicle-mediated transport | 0.39 | 0.011 |
| BP_immune response-activating signal transduction | 0.44 | 0.011 |
| BP_regulation of cell death | 0.24 | 0.012 |
| BP_acute inflammatory response | 0.44 | 0.012 |
| BP_immune system process | 0.21 | 0.012 |
| BP_regulation of multicellular organismal development | 0.22 | 0.012 |
| BP_cell communication | 0.18 | 0.012 |
| BP_macromolecule metabolic process | 0.18 | 0.012 |
| BP_regulation of cell development | 0.28 | 0.012 |
| BP_lipid catabolic process | -0.40 | 0.012 |
| BP_oxoacid metabolic process | -0.25 | 0.013 |
| BP_alpha-amino acid metabolic process | -0.36 | 0.013 |
| BP_nuclear division | 0.37 | 0.013 |
| BP_embryonic organ morphogenesis | 0.41 | 0.013 |
| BP_transport | 0.19 | 0.013 |
| BP_positive regulation of protein modification process | 0.26 | 0.013 |
| BP_regulation of primary metabolic process | 0.20 | 0.014 |
| BP_negative regulation of protein kinase activity | 0.53 | 0.014 |
| BP_germ cell development | 0.54 | 0.014 |
| BP_cellular response to insulin stimulus | 0.49 | 0.014 |
| BP_immune response-activating cell surface receptor signaling pathway | 0.61 | 0.014 |
| BP_cellular lipid catabolic process | -0.44 | 0.014 |
| BP_JAK-STAT cascade | 0.60 | 0.014 |
| BP_regulation of JAK-STAT cascade | 0.60 | 0.014 |
| BP_STAT cascade | 0.60 | 0.014 |
| BP_regulation of STAT cascade | 0.60 | 0.014 |
| BP_oxidation-reduction process | -0.25 | 0.014 |
| BP_positive regulation of programmed cell death | 0.37 | 0.014 |
| BP_regulation of systemic arterial blood pressure | -0.48 | 0.015 |
| BP_regulation of protein serine/threonine kinase activity | 0.37 | 0.015 |
| BP_localization | 0.18 | 0.015 |
| BP_response to insulin | 0.44 | 0.015 |
| BP_response to drug | -0.23 | 0.015 |
| BP_response to nutrient levels | -0.34 | 0.015 |
| BP_coenzyme metabolic process | -0.40 | 0.015 |
| BP_regulation of body fluid levels | 0.31 | 0.016 |
| BP_sensory organ morphogenesis | 0.40 | 0.016 |
| BP_immune response-regulating cell surface receptor signaling pathway | 0.53 | 0.017 |
| BP_cellular process involved in reproduction in multicellular organism | 0.42 | 0.018 |
| BP_ion transport | -0.20 | 0.018 |
| BP_establishment of localization | 0.18 | 0.019 |
| BP_monocarboxylic acid transport | -0.42 | 0.019 |
| BP_endocytosis | 0.34 | 0.019 |
| BP_protein secretion | 0.31 | 0.019 |
| BP_proteolysis | 0.24 | 0.019 |
| BP_locomotion | 0.22 | 0.019 |
| BP_monosaccharide metabolic process | -0.47 | 0.019 |
| BP_cellular developmental process | 0.19 | 0.019 |
| BP_myeloid cell differentiation | 0.47 | 0.019 |
| BP_negative regulation of kinase activity | 0.50 | 0.019 |
| BP_protein phosphorylation | 0.23 | 0.020 |
| BP_regulation of immune response | 0.30 | 0.021 |
| BP_sister chromatid segregation | 0.42 | 0.022 |
| BP_positive regulation of locomotion | 0.29 | 0.022 |
| BP_mitotic spindle organization | 0.52 | 0.022 |
| BP_negative regulation of metabolic process | 0.22 | 0.022 |
| BP_cellular response to xenobiotic stimulus | -0.38 | 0.023 |
| BP_positive regulation of metabolic process | 0.20 | 0.023 |
| BP_multicellular organism development | 0.18 | 0.023 |
| BP_defense response to bacterium | 0.42 | 0.023 |
| BP_organelle organization | 0.20 | 0.023 |
| BP_regulation of protein modification process | 0.24 | 0.023 |
| BP_peptide secretion | 0.30 | 0.024 |
| BP_excretion | 0.49 | 0.025 |
| BP_activation of immune response | 0.39 | 0.025 |
| BP_movement of cell or subcellular component | 0.21 | 0.025 |
| BP_regulation of neurotransmitter levels | -0.33 | 0.026 |
| BP_regulation of nervous system development | 0.28 | 0.026 |
| BP_regulation of signaling | 0.19 | 0.026 |
| BP_amino acid transmembrane transport | -0.45 | 0.026 |
| BP_regulation of cell cycle process | 0.30 | 0.027 |
| BP_cellular macromolecule catabolic process | 0.33 | 0.027 |
| BP_extracellular matrix organization | 0.32 | 0.027 |
| BP_regulation of peptidase activity | 0.35 | 0.028 |
| BP_hexose metabolic process | -0.50 | 0.028 |
| BP_regulation of molecular function | 0.20 | 0.028 |
| BP_regulation of lipid localization | -0.47 | 0.029 |
| BP_appendage morphogenesis | 0.52 | 0.029 |
| BP_limb morphogenesis | 0.52 | 0.029 |
| BP_negative regulation of cell cycle | 0.34 | 0.029 |
| BP_system development | 0.18 | 0.029 |
| BP_activation of protein kinase activity | 0.41 | 0.030 |
| BP_regulation of cell communication | 0.19 | 0.030 |
| BP_regulation of proteolysis | 0.31 | 0.030 |
| BP_oxidoreduction coenzyme metabolic process | -0.49 | 0.031 |
| BP_pyridine nucleotide metabolic process | -0.49 | 0.031 |
| BP_nicotinamide nucleotide metabolic process | -0.49 | 0.031 |
| BP_peptidyl-amino acid modification | 0.27 | 0.031 |
| BP_negative regulation of transferase activity | 0.45 | 0.031 |
| BP_extrinsic apoptotic signaling pathway | 0.45 | 0.031 |
| BP_cell cycle checkpoint | 0.45 | 0.031 |
| BP_digestion | -0.38 | 0.032 |
| BP_regulation of phosphate metabolic process | 0.23 | 0.032 |
| BP_regulation of phosphorus metabolic process | 0.23 | 0.032 |
| BP_positive regulation of protein phosphorylation | 0.26 | 0.032 |
| BP_regulation of transport | 0.23 | 0.032 |
| BP_regulation of peptidyl-tyrosine phosphorylation | 0.38 | 0.033 |
| BP_regulation of neurogenesis | 0.27 | 0.033 |
| BP_amino acid transport | -0.37 | 0.033 |
| BP_regulation of localization | 0.20 | 0.033 |
| BP_mitotic sister chromatid segregation | 0.42 | 0.033 |
| BP_humoral immune response | 0.44 | 0.033 |
| BP_leukocyte migration | 0.28 | 0.034 |
| BP_response to extracellular stimulus | -0.31 | 0.034 |
| BP_defense response to other organism | 0.37 | 0.034 |
| BP_microtubule cytoskeleton organization involved in mitosis | 0.48 | 0.034 |
| BP_inorganic anion transport | 0.39 | 0.034 |
| BP_positive regulation of establishment of protein localization | 0.36 | 0.035 |
| BP_axon guidance | 0.42 | 0.035 |
| BP_neuron projection guidance | 0.42 | 0.035 |
| BP_embryonic organ development | 0.33 | 0.035 |
| BP_nervous system development | 0.21 | 0.035 |
| BP_phosphorylation | 0.21 | 0.036 |
| BP_regulation of extrinsic apoptotic signaling pathway | 0.47 | 0.036 |
| BP_chromatin organization | 0.37 | 0.036 |
| BP_membrane organization | 0.27 | 0.036 |
| BP_cell cycle phase transition | 0.30 | 0.037 |
| BP_sensory perception of sound | 0.46 | 0.037 |
| BP_sensory perception of mechanical stimulus | 0.46 | 0.037 |
| BP_import into cell | 0.29 | 0.038 |
| BP_regulation of phosphorylation | 0.23 | 0.038 |
| BP_negative regulation of phosphorylation | 0.33 | 0.039 |
| BP_nitrogen compound transport | 0.21 | 0.039 |
| BP_leukocyte chemotaxis | 0.38 | 0.039 |
| BP_nuclear chromosome segregation | 0.38 | 0.039 |
| BP_DNA conformation change | 0.50 | 0.039 |
| BP_DNA repair | 0.49 | 0.039 |
| BP_negative regulation of peptidase activity | 0.38 | 0.039 |
| BP_cellular cation homeostasis | 0.30 | 0.040 |
| BP_spindle assembly | 0.52 | 0.040 |
| BP_positive regulation of axonogenesis | 0.53 | 0.040 |
| BP_regulation of anatomical structure morphogenesis | 0.25 | 0.040 |
| BP_response to xenobiotic stimulus | -0.33 | 0.041 |
| BP_cellular response to chemical stimulus | 0.19 | 0.041 |
| BP_mitotic cell cycle checkpoint | 0.45 | 0.042 |
| BP_synapse assembly | 0.44 | 0.042 |
| BP_positive regulation of transport | 0.25 | 0.042 |
| BP_positive regulation of cell morphogenesis involved in differentiation | 0.43 | 0.042 |
| BP_innate immune response | 0.29 | 0.042 |
| BP_regulation of neuron projection development | 0.33 | 0.042 |
| BP_negative regulation of nitrogen compound metabolic process | 0.22 | 0.043 |
| BP_negative regulation of neurogenesis | 0.41 | 0.043 |
| BP_negative regulation of nervous system development | 0.41 | 0.043 |
| BP_dicarboxylic acid transport | -0.42 | 0.043 |
| BP_positive regulation of kinase activity | 0.29 | 0.043 |
| BP_regulation of endocytosis | 0.43 | 0.043 |
| BP_regulation of signal transduction | 0.19 | 0.044 |
| BP_immune response | 0.22 | 0.044 |
| BP_positive regulation of immune response | 0.32 | 0.044 |
| BP_regulation of protein transport | 0.29 | 0.044 |
| BP_regulation of peptide transport | 0.29 | 0.044 |
| BP_collagen metabolic process | 0.47 | 0.044 |
| BP_positive regulation of protein transport | 0.35 | 0.045 |
| BP_regulation of protein phosphorylation | 0.23 | 0.045 |
| BP_regulation of cellular catabolic process | 0.34 | 0.045 |
| BP_nucleotide metabolic process | -0.29 | 0.046 |
| BP_positive regulation of cell cycle | 0.36 | 0.046 |
| BP_embryonic morphogenesis | 0.28 | 0.046 |
| BP_regulation of endopeptidase activity | 0.35 | 0.046 |
| BP_myeloid leukocyte differentiation | 0.47 | 0.046 |
| BP_secretion | 0.21 | 0.046 |
| BP_negative regulation of phosphorus metabolic process | 0.31 | 0.046 |
| BP_negative regulation of phosphate metabolic process | 0.31 | 0.046 |
| BP_cellular modified amino acid metabolic process | -0.32 | 0.047 |
| BP_positive regulation of molecular function | 0.22 | 0.047 |
| BP_DNA metabolic process | 0.32 | 0.048 |
| BP_anion transmembrane transport | -0.29 | 0.048 |
| BP_secretion by cell | 0.22 | 0.048 |
| BP_anaphase-promoting complex-dependent catabolic process | 0.51 | 0.049 |
| BP_negative regulation of growth | 0.42 | 0.049 |
| CC_protein-containing complex | 0.26 | 0.001 |
| CC_basolateral plasma membrane | 0.43 | 0.002 |
| CC_brush border membrane | -0.69 | 0.002 |
| CC_cluster of actin-based cell projections | -0.61 | 0.002 |
| CC_brush border | -0.67 | 0.002 |
| CC_cell projection membrane | -0.42 | 0.003 |
| CC_apical plasma membrane | -0.34 | 0.003 |
| CC_membrane-enclosed lumen | 0.22 | 0.003 |
| CC_organelle lumen | 0.22 | 0.003 |
| CC_intracellular organelle lumen | 0.22 | 0.003 |
| CC_apical part of cell | -0.34 | 0.003 |
| CC_plasma membrane region | -0.24 | 0.003 |
| CC_membrane protein complex | 0.38 | 0.003 |
| CC_extracellular organelle | -0.21 | 0.004 |
| CC_extracellular vesicle | -0.21 | 0.004 |
| CC_plasma membrane part | -0.21 | 0.004 |
| CC_extracellular exosome | -0.21 | 0.004 |
| CC_extracellular space | -0.22 | 0.004 |
| CC_integral component of membrane | -0.19 | 0.004 |
| CC_extracellular region part | -0.18 | 0.004 |
| CC_intrinsic component of membrane | -0.19 | 0.004 |
| CC_membrane part | -0.20 | 0.004 |
| CC_nucleus | 0.22 | 0.004 |
| CC_nuclear part | 0.23 | 0.004 |
| CC_extracellular matrix | 0.33 | 0.005 |
| CC_collagen-containing extracellular matrix | 0.36 | 0.005 |
| CC_endoplasmic reticulum membrane | -0.31 | 0.005 |
| CC_nuclear outer membrane-endoplasmic reticulum membrane network | -0.31 | 0.005 |
| CC_endoplasmic reticulum subcompartment | -0.31 | 0.005 |
| CC_chromosomal region | 0.47 | 0.005 |
| CC_condensed chromosome | 0.47 | 0.007 |
| CC_plasma membrane | -0.18 | 0.008 |
| CC_cell projection part | -0.25 | 0.009 |
| CC_plasma membrane bounded cell projection part | -0.25 | 0.009 |
| CC_organelle part | 0.18 | 0.009 |
| CC_chromosome | 0.34 | 0.010 |
| CC_nuclear lumen | 0.23 | 0.012 |
| CC_cell periphery | -0.17 | 0.012 |
| CC_cell projection | -0.20 | 0.012 |
| CC_endosomal part | 0.41 | 0.014 |
| CC_endosome membrane | 0.44 | 0.014 |
| CC_condensed chromosome, centromeric region | 0.50 | 0.015 |
| CC_plasma membrane bounded cell projection | -0.21 | 0.016 |
| CC_midbody | 0.47 | 0.016 |
| CC_ion channel complex | 0.46 | 0.017 |
| CC_transmembrane transporter complex | 0.46 | 0.017 |
| CC_transporter complex | 0.46 | 0.017 |
| CC_integral component of plasma membrane | -0.22 | 0.019 |
| CC_blood microparticle | 0.51 | 0.019 |
| CC_intrinsic component of plasma membrane | -0.21 | 0.022 |
| CC_intracellular organelle part | 0.18 | 0.024 |
| CC_chromosome, centromeric region | 0.45 | 0.026 |
| CC_endoplasmic reticulum lumen | 0.33 | 0.027 |
| CC_collagen trimer | 0.46 | 0.028 |
| CC_chromosomal part | 0.33 | 0.029 |
| CC_vesicle | -0.16 | 0.031 |
| CC_nucleoplasm | 0.22 | 0.031 |
| CC_cytoplasmic vesicle part | 0.24 | 0.032 |
| CC_cytoplasmic vesicle | 0.21 | 0.035 |
| CC_intracellular vesicle | 0.21 | 0.035 |
| CC_intracellular membrane-bounded organelle | 0.17 | 0.041 |
| CC_recycling endosome | 0.50 | 0.042 |
| CC_kinetochore | 0.48 | 0.045 |
| CC_condensed chromosome kinetochore | 0.48 | 0.045 |
| CC_cell cortex | 0.42 | 0.049 |
| MF_ion channel activity | 0.43 | 0.002 |
| MF_chloride transmembrane transporter activity | 0.78 | 0.002 |
| MF_anion channel activity | 0.74 | 0.002 |
| MF_peptide receptor activity | -0.63 | 0.002 |
| MF_G protein-coupled peptide receptor activity | -0.63 | 0.002 |
| MF_solute:sodium symporter activity | -0.73 | 0.002 |
| MF_monooxygenase activity | -0.60 | 0.002 |
| MF_solute:cation symporter activity | -0.71 | 0.002 |
| MF_sodium ion transmembrane transporter activity | -0.58 | 0.002 |
| MF_symporter activity | -0.61 | 0.003 |
| MF_monovalent inorganic cation transmembrane transporter activity | -0.44 | 0.003 |
| MF_organic anion transmembrane transporter activity | -0.37 | 0.003 |
| MF_metal ion transmembrane transporter activity | -0.43 | 0.003 |
| MF_secondary active transmembrane transporter activity | -0.55 | 0.003 |
| MF_inorganic cation transmembrane transporter activity | -0.41 | 0.003 |
| MF_active transmembrane transporter activity | -0.53 | 0.003 |
| MF_cation transmembrane transporter activity | -0.37 | 0.003 |
| MF_transmembrane transporter activity | -0.30 | 0.003 |
| MF_transporter activity | -0.28 | 0.003 |
| MF_inorganic molecular entity transmembrane transporter activity | -0.31 | 0.003 |
| MF_ion transmembrane transporter activity | -0.30 | 0.003 |
| MF_substrate-specific channel activity | 0.42 | 0.003 |
| MF_enzyme inhibitor activity | 0.43 | 0.003 |
| MF_serine-type endopeptidase inhibitor activity | 0.61 | 0.004 |
| MF_anion transmembrane transporter activity | -0.35 | 0.006 |
| MF_channel activity | 0.39 | 0.006 |
| MF_oxidoreductase activity, acting on paired donors, with incorporation or reduction of molecular oxygen | -0.50 | 0.007 |
| MF_organic acid transmembrane transporter activity | -0.45 | 0.007 |
| MF_catalytic activity | -0.18 | 0.008 |
| MF_passive transmembrane transporter activity | 0.37 | 0.008 |
| MF_extracellular matrix structural constituent | 0.43 | 0.009 |
| MF_kinase regulator activity | 0.57 | 0.009 |
| MF_protein kinase regulator activity | 0.57 | 0.009 |
| MF_growth factor receptor binding | 0.56 | 0.009 |
| MF_lyase activity | -0.48 | 0.014 |
| MF_carboxylic acid transmembrane transporter activity | -0.42 | 0.014 |
| MF_cytokine receptor binding | 0.46 | 0.014 |
| MF_inorganic anion transmembrane transporter activity | 0.43 | 0.016 |
| MF_serine-type endopeptidase activity | 0.46 | 0.018 |
| MF_amino acid transmembrane transporter activity | -0.49 | 0.018 |
| MF_heparin binding | 0.42 | 0.019 |
| MF_RNA polymerase II regulatory region sequence-specific DNA binding | 0.36 | 0.019 |
| MF_RNA polymerase II regulatory region DNA binding | 0.36 | 0.019 |
| MF_sequence-specific double-stranded DNA binding | 0.34 | 0.021 |
| MF_transcription regulatory region sequence-specific DNA binding | 0.34 | 0.022 |
| MF_regulatory region nucleic acid binding | 0.33 | 0.022 |
| MF_transcription regulatory region DNA binding | 0.33 | 0.022 |
| MF_tetrapyrrole binding | -0.49 | 0.025 |
| MF_enzyme regulator activity | 0.28 | 0.030 |
| MF_sequence-specific DNA binding | 0.30 | 0.033 |
| MF_heme binding | -0.49 | 0.033 |
| MF_cofactor binding | -0.29 | 0.036 |
| MF_structural molecule activity | 0.29 | 0.038 |
| MF_glycosaminoglycan binding | 0.35 | 0.038 |
| MF_double-stranded DNA binding | 0.32 | 0.040 |
| MF_serine-type peptidase activity | 0.42 | 0.043 |
| MF_serine hydrolase activity | 0.42 | 0.043 |
| MF_sulfur compound binding | 0.35 | 0.045 |

# Supplementary Table 9 Enriched KEGG term in validation group.

| Description | enrichmentScore | pvalue |
| --- | --- | --- |
| Phagosome | 0.58 | 0.002 |
| Vibrio cholerae infection | 0.80 | 0.002 |
| p53 signaling pathway | 0.66 | 0.002 |
| Bile secretion | -0.50 | 0.002 |
| Rheumatoid arthritis | 0.54 | 0.007 |
| Axon guidance | 0.53 | 0.009 |
| Breast cancer | 0.60 | 0.011 |
| Metabolic pathways | -0.21 | 0.012 |
| Neuroactive ligand-receptor interaction | -0.34 | 0.014 |
| Human papillomavirus infection | 0.37 | 0.023 |
| Cellular senescence | 0.51 | 0.025 |
| Cell cycle | 0.45 | 0.044 |
| MicroRNAs in cancer | 0.46 | 0.044 |
| Ras signaling pathway | 0.40 | 0.071 |
| Platelet activation | 0.43 | 0.113 |
| MAPK signaling pathway | 0.31 | 0.134 |
| Gastric cancer | 0.41 | 0.149 |
| Coronavirus disease - COVID-19 | 0.37 | 0.162 |
| Phospholipase D signaling pathway | 0.34 | 0.189 |
| Prostate cancer | 0.40 | 0.196 |
| Parkinson disease | -0.39 | 0.222 |
| Pathways in cancer | 0.22 | 0.271 |
| Human T-cell leukemia virus 1 infection | 0.30 | 0.275 |
| Transcriptional misregulation in cancer | 0.33 | 0.296 |
| Lipid and atherosclerosis | 0.36 | 0.299 |
| Gastric acid secretion | 0.37 | 0.299 |
| Regulation of actin cytoskeleton | 0.33 | 0.303 |
| Hippo signaling pathway | 0.33 | 0.347 |
| Protein digestion and absorption | -0.25 | 0.351 |
| cAMP signaling pathway | -0.31 | 0.366 |
| Cell adhesion molecules | 0.27 | 0.373 |
| Oocyte meiosis | 0.30 | 0.400 |
| Progesterone-mediated oocyte maturation | 0.31 | 0.434 |
| Insulin resistance | -0.27 | 0.450 |
| PI3K-Akt signaling pathway | 0.21 | 0.485 |
| Complement and coagulation cascades | 0.27 | 0.496 |
| Dilated cardiomyopathy | -0.27 | 0.516 |
| Proteoglycans in cancer | 0.24 | 0.522 |
| Alzheimer disease | -0.24 | 0.528 |
| Rap1 signaling pathway | 0.23 | 0.567 |
| HIF-1 signaling pathway | 0.26 | 0.582 |
| FoxO signaling pathway | -0.24 | 0.593 |
| AGE-RAGE signaling pathway in diabetic complications | 0.26 | 0.614 |
| AMPK signaling pathway | -0.25 | 0.627 |
| Biosynthesis of amino acids | -0.25 | 0.629 |
| Growth hormone synthesis, secretion and action | 0.27 | 0.630 |
| Hepatocellular carcinoma | 0.23 | 0.664 |
| Human cytomegalovirus infection | 0.24 | 0.673 |
| Wnt signaling pathway | 0.26 | 0.678 |
| Relaxin signaling pathway | 0.25 | 0.694 |
| Pathways of neurodegeneration - multiple diseases | -0.19 | 0.735 |
| Calcium signaling pathway | 0.18 | 0.769 |
| JAK-STAT signaling pathway | 0.23 | 0.777 |
| Cytokine-cytokine receptor interaction | -0.17 | 0.810 |
| Glutathione metabolism | 0.22 | 0.827 |
| Viral protein interaction with cytokine and cytokine receptor | 0.21 | 0.841 |
| Insulin secretion | 0.22 | 0.842 |
| Biosynthesis of cofactors | -0.18 | 0.846 |
| Glutamatergic synapse | 0.21 | 0.848 |
| Purine metabolism | -0.19 | 0.860 |
| Prion disease | 0.21 | 0.862 |
| IL-17 signaling pathway | 0.19 | 0.863 |
| Hypertrophic cardiomyopathy | -0.20 | 0.865 |
| Chemical carcinogenesis - receptor activation | 0.16 | 0.890 |
| ECM-receptor interaction | -0.16 | 0.895 |
| Choline metabolism in cancer | 0.20 | 0.900 |
| Vascular smooth muscle contraction | 0.19 | 0.915 |
| Fluid shear stress and atherosclerosis | 0.18 | 0.917 |
| Drug metabolism - other enzymes | -0.16 | 0.927 |
| Melanogenesis | 0.17 | 0.964 |
| Cushing syndrome | -0.14 | 0.972 |
| Chemokine signaling pathway | 0.12 | 0.982 |
| Diabetic cardiomyopathy | -0.13 | 0.984 |
| TNF signaling pathway | 0.14 | 0.986 |
| Focal adhesion | -0.12 | 0.988 |
| Chemical carcinogenesis - reactive oxygen species | -0.13 | 0.993 |
| Adrenergic signaling in cardiomyocytes | 0.12 | 1.000 |
